# Flexible multitask computation in recurrent networks utilizes shared dynamical motifs

**DOI:** 10.1038/s41593-024-01668-6

**Published:** 2024-07-09

**Authors:** Laura N. Driscoll, Krishna Shenoy, David Sussillo

**Affiliations:** 1 Department of Electrical Engineering, Stanford University, Stanford, CA USA; 2 Department of Neurosurgery, Stanford University, Stanford, CA USA; 3 Department of Bioengineering, Stanford University, Stanford, CA USA; 4 Department of Neurobiology, Stanford University, Stanford, CA USA; 5 Wu Tsai Neurosciences Institute, Stanford University, Stanford, CA USA; 6 Bio-X Institute, Stanford University, Stanford, CA USA; 7 Howard Hughes Medical Institute at Stanford University, Stanford, CA USA

**Keywords:** Dynamical systems, Decision

## Abstract

Flexible computation is a hallmark of intelligent behavior. However, little is known about how neural networks contextually reconfigure for different computations. In the present work, we identified an algorithmic neural substrate for modular computation through the study of multitasking artificial recurrent neural networks. Dynamical systems analyses revealed learned computational strategies mirroring the modular subtask structure of the training task set. Dynamical motifs, which are recurring patterns of neural activity that implement specific computations through dynamics, such as attractors, decision boundaries and rotations, were reused across tasks. For example, tasks requiring memory of a continuous circular variable repurposed the same ring attractor. We showed that dynamical motifs were implemented by clusters of units when the unit activation function was restricted to be positive. Cluster lesions caused modular performance deficits. Motifs were reconfigured for fast transfer learning after an initial phase of learning. This work establishes dynamical motifs as a fundamental unit of compositional computation, intermediate between neuron and network. As whole-brain studies simultaneously record activity from multiple specialized systems, the dynamical motif framework will guide questions about specialization and generalization.

## Abstract (web summary)

The authors identify reusable ‘dynamical motifs’ in artificial neural networks. These motifs enable flexible recombination of previously learned capabilities, promoting modular, compositional computation and rapid transfer learning. This discovery sheds light on the fundamental building blocks of intelligent behavior.

## Main

Cognitive flexibility is a key feature of the human brain. Although artificial systems are capable of outperforming humans in specific tasks^1–3^, they so far lack flexibility for rapid learning and task switching. A major open question in the fields of neuroscience and artificial intelligence is how the same circuit reconfigures to perform multiple tasks.

Conceptual models for cognitive flexibility propose a hierarchy of elementary processes that are reused across similar tasks^4–6^. According to these models, the neural substrate for computation is modular such that combinations of previously learned subtasks may be reconfigured to perform unfamiliar tasks. This combination of subtasks is referred to as compositionality^6^. For example, a saccade task typically involves a cue that indicates in which direction to move the eyes. After learning a saccade task, a person could quickly learn an ‘anti’ version of the same task where the same cue now instructs a saccade in the opposite direction. This new task may be quickly learned by combining a computational building block for the original task with a previously learned ‘anti’ building block. Although there is some experimental evidence that neural computation is compositional^7,8^, a concrete model for its implementation hinges on identifying modular components with compositional potential. Although the time and effort required to train animals to perform many tasks has limited the exploration of multitask computation in biological networks, artificial neural networks now present an opportunity to explore the topic. The study of cognition through simulations in artificial networks has led to substantial advances in understanding neural computation in the past decade^9–19^. However, researchers typically trained artificial neural networks to perform single tasks in isolation, with few exceptions^20–25^, limiting the insights into biological neural circuits that perform many tasks. One exception to this trend is the study by Yang et al.^21^, in which the authors trained a single network to perform 20 related tasks and, thereby, identified clustered representations in state space that supported task compositionality. In the present work, we identified the computational substrate that allowed for modular computation in these networks, which we call ‘dynamical motifs’. These motifs are distinctive features of the dynamics that reoccur across different implementations of similar computations.

We examined multitask networks through the lens of dynamical systems. This approach allowed us to explore the mechanisms underlying computation in a recurrently connected artificial network^26^. We found that tasks that required the same computational elements (for example, memory, categorization and delayed response) were implemented by sharing and repurposing dynamical motifs (for example, attractors, decision boundaries and rotations).

## Results

### Network structure

**Fig. 1:**
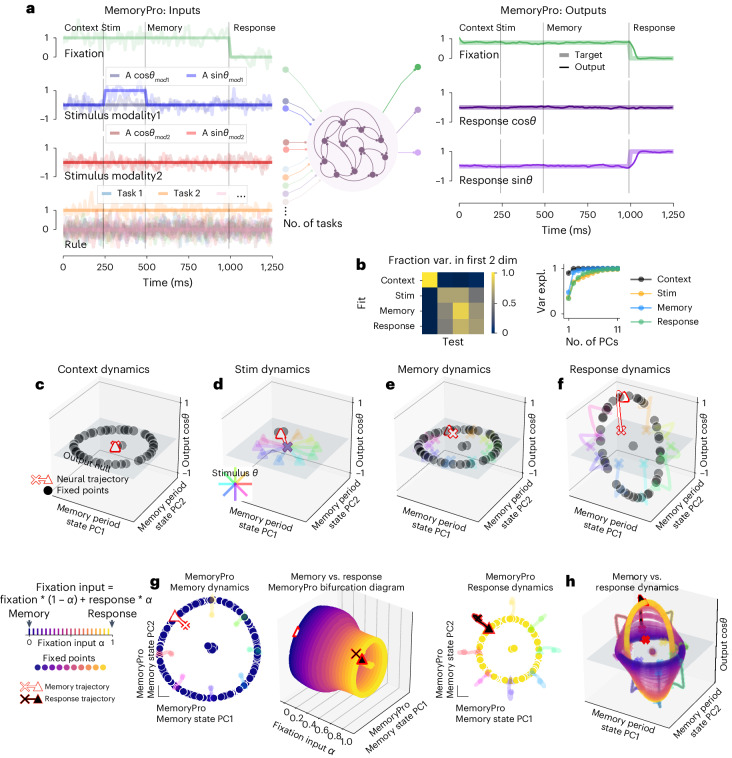
Single-task network shared fixed points across task periods. **a**, Left: noisy fixation, stimulus (modality 1 and modality 2) and rule input time series (overlayed without noise for clarity). Noise was used during training, and analyses were performed on running the network without noise. Vertical lines divide task periods: context, stimulus, memory and response. Right: targets (thick lines) overlaid with outputs of a trained network (thin lines). Stim, stimulus task period. **b**, Fraction variance explained in each task period by top two PCs of neural state trajectories for 1,024 stimulus conditions from every other task period (left). We performed PCA on task period Y and then calculated how much variance it explained on task period X. Right: top 11 PCs of neural state trajectories for 1,024 stimulus conditions from each task period. **c**–**f**, State space plots for single-task network performing MemoryPro during context (**c**), stimulus (**d**), memory (**e**) and response (**f**) task periods. State trajectories and fixed points projected onto the first two PCs defined by state trajectories during the memory period on the *x* and *y* axes and the output weight vector (from *W*_*ou*t_) associated with cos*θ*_stimulus_ on the *z* axis. We visualized fixed point locations for *θ*_stimulus_ = *0* (black dots) in all subpanels of Fig. 1 and additionally plotted state trajectories for other stimulus conditions (see 'Fixed points' in Methods for further details on fixed point identification). State trajectories (colored lines) are colored according to stimulus orientation with *θ*_stimulus_ = 0 highlighted in red, starting from ‘x’ and ending with ‘▴’. **g**, Interpolation between inputs for memory (*α* *=* 0) and response (*α* *=* 1) periods. Middle: fixed points for 20 intermediate *α* values (*x* axis) projected into top two memory period PCs (as in **c**–**f**) (*y* and *z* axes) with memory *α* *=* 0 (left) and response *α* *=* 1 (right) fixed points and trajectories. **h**, Fixed points for input interpolation between memory (blue) and response (yellow) inputs. State trajectories are colored according to stimulus orientation. Same axes as **c**–**f**. All subpanels were generated from the same network with hyperparameters: 256 units, softplus activation, diagonal initialization.

**Extended Data Fig. 1:**
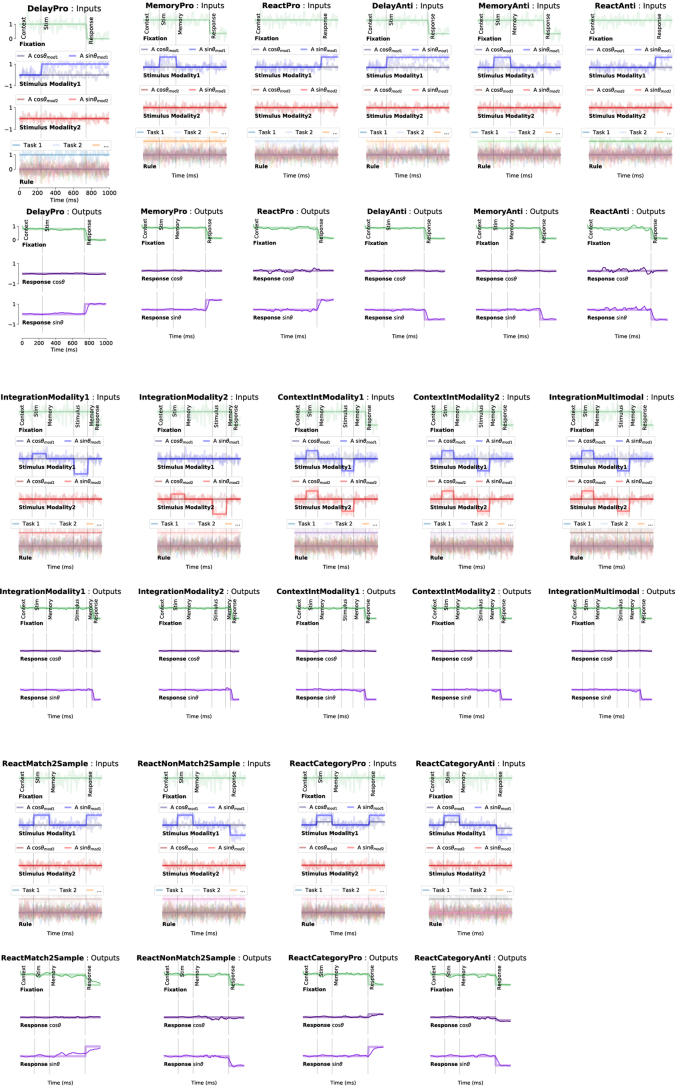
Inputs and outputs for each task for one example trial. One panel for each task. **Top:** Noisy fixation, stimulus (modality 1 and 2), and rule input time-series (overlayed without noise for clarity). Noise was used during training while analyses were performed by running the network without noise (except Fig. 4b, which was run with noise). Vertical lines divide task periods. **Bottom:** Targets (thick lines) overlaid with outputs of a trained network (thin lines).

We implemented a similar input–output structure and learning protocol as in previously examined multitasking recurrent neural networks (RNNs)^21^. These tasks included reaction-timed, delayed response and memory tasks with contextual integration, categorization, pro response and anti response components (see Extended Data Fig. 1, Supplementary Table 1 and 'Tasks and performances' in Methods for task definitions). For every task, the network received three noisy inputs: fixation (one-dimensional), stimulus (four-dimensional) and rule (15-dimensional) (Fig. 1a). The fixation input directed the network to either output zero or respond. The set of stimuli contained two separate two-dimensional vectors composed of *A*sin*θ* and *A*cos*θ*, where each vector encoded a different one-dimensional circular variable (*θ*_1_, *θ*_2_) scaled by an amplitude (*A*_1_, *A*_2_). Depending on the rule, one stimulus vector may be contextually ignored. The rule input indicated the current task on any given trial, and this information was continuously available to the network throughout each trial. Rule input was encoded in a one-hot vector where the index associated with the current task was 1 and all other indices were 0.

The RNN is defined by

1 $$\tau {\frac{{\rm{d}}{\bf{h}}}{{\rm{d}}t}}=-{\bf{h}}(t)+\sigma ({W}_{{\rm{rec}}}{\bf{h}}(t)+{W}_{{\rm{in}}}{\bf{u}}(t)+{{\bf{b}}}_{\rm{in}}+{\bf{\upxi}} (t))$$

2 $${\bf{z}}\left(t\right)={W}_{{{\rm{out}}}}{\bf{h}}\left(t\right)+{{\bf{b}}}_{{{\rm{out}}}}$$

3 $$\sigma \left({\bf{h}}\right)=\mathrm{ln}\left(1+\exp \left({\bf{h}}\right)\right)$$

All inputs, **u**(*t*) (20 × 1), enter the system and induce a specific pattern of activity, **h**(*t*) (*N*_rec_ × 1), in the units of the RNN (equation (1)). We refer to this *N*_rec_*-*dimensional vector, **h**(*t*), as the state of the network at time *t*. There was noise in the inputs and independent noise in each unit, ${\bf{\upxi}} (t)$. The output, **z**(*t*) (3 × 1), is a linear projection of the state (equation (2)). The output units indicate whether the network is responding in the first dimension and in which direction on a circle the RNN responds in the next two dimensions (for example, saccade direction) (Fig. 1a, right). For consistency, in most of this paper, we will focus on RNNs as described by equation (1), using diagonal initialization of *W*_rec_, the softplus nonlinear activation function (equation (3)) and L2 activity and weight regularization. We identified shared dynamical motifs in all explored network designs and include comparisons to other parameter choices throughout. All network weights were trained to minimize the squared difference between the network output and a desired target using back propagation through time.

Our approach was to uncover the underlying learned dynamical systems in trained RNNs to mechanistically understand how networks implement computation. This approach utilizes fixed points of equation (1) to provide an interpretable ‘skeleton’ of the complex high-dimensional dynamics^26–28^. By studying how fixed points change as a function of the inputs, we may understand if and how fixed point structures are repurposed for different computations. In the absence of noise, inputs to the network were piecewise constant, where every change in the inputs marked the beginning of a new task period (Fig. 1a, vertical lines). Therefore, during each task period with unique inputs (for example, stimulus, context/memory and response), the network could be treated as a separate, autonomous dynamical system with a distinct set of fixed points from other task periods. Going forward, we use ‘dynamical motif’ to mean the high-dimensional nonlinear dynamics around a fixed point skeleton that implements computation for a specified input. See Methods for further details on network setup, training and fixed point analysis.

### Single-task networks

We first trained individual networks to perform each task in isolation. For example, in a MemoryPro task, the network should respond in the same direction as the stimulus after a memory period. There were four periods (visually divided by vertical lines Fig. 1a). We quantified the fraction of state trajectory variance explained in the top 10 principal components (PCs) for a given task period and in the top two PCs defined by every other task period (Fig. 1b). This provides a reference for the fraction of variance captured in visualizations (Fig. 1c–h). We visualized the high-dimensional network state trajectories and fixed points in a low-dimensional PC space defined by performing principal component analysis (PCA) on state trajectories during the memory period of the MemoryPro task. In the first period (context), the rule input indicated which task the network performed for that trial. In a network trained to perform only the MemoryPro task, the context period inputs result in one fixed point at the center of a ring of fixed points (Fig. 1c). The central fixed point serves as an initial condition for performing the task computation during the ensuing stimulus period. Notice that the context period inputs are identical to the memory period (rule and fixation on, stimulus off) (Fig. 1a), so the fixed points are necessarily identical between these task periods. We show later that the additional ring of fixed points was relevant to the memory computation during the memory task period.

In the stimulus period, we examined the fixed point structure for each stimulus input separately. Stimulus period state trajectories for different stimuli diverged from the central initial condition toward stimulus-dependent fixed points, mapping out a stimulus representation that was orthogonal (null) to the response readout dimension (Fig. 1d). During the memory period, the state evolved toward a ring of fixed points that made up an approximate ring attractor (locally attracting structure in all dimensions except tangent to the ring, which is neither contracting nor expanding) (Fig. 1e). Although fixed points were identical in the context and memory periods, the network state interacted with different fixed points due to different initial conditions. Together, these fixed points stored the identity of the stimulus orientation based on the initial conditions of the state at the beginning of the memory period (end of the stimulus period). During the response period, the fixation input changed to zero, and a new ring attractor emerged (Fig. 1f). During the response period, the ring was oriented such that it had a non-zero dot product with the output weights (*W*_out_) and was, therefore, output potent^29^. The new ring caused the network to respond in the appropriate orientation based on the initial conditions of the response period (end of the memory period). Thus, the network responded with the appropriate orientation for this task (*φ*_response_ *=* *θ*_stimulus_; see Methods for definitions of all tasks).

What is the relationship between the ring of fixed points in the memory and response periods? To address this question, we traced locations of the fixed points during interpolation across memory and response period inputs, (1 *−* *α*)**u**_memory_ + *α***u**_response_, where *α* was incremented in 0.05 steps between 0 and 1. We identified fixed points for each incremental input setting as a function of *α* ('Input interpolation' in Methods). By interpolating across input conditions for the memory and response periods, we traced how fixed points moved and changed stability as the dynamical system reconfigured.

For every intermediate input value throughout interpolation, an approximate ring attractor was present (Fig. 1g). The smooth transition of this fixed point structure implies that each intermediate ring attractor was functionally the same ring attractor across input conditions. In this single-task network, the dynamical motif that performed memory and response computations was shared across task periods. The ring attractor rotated from output null space into output potent space when the fixation input changed to zero (Fig. 1h).

### Two-task networks

**Fig. 2:**
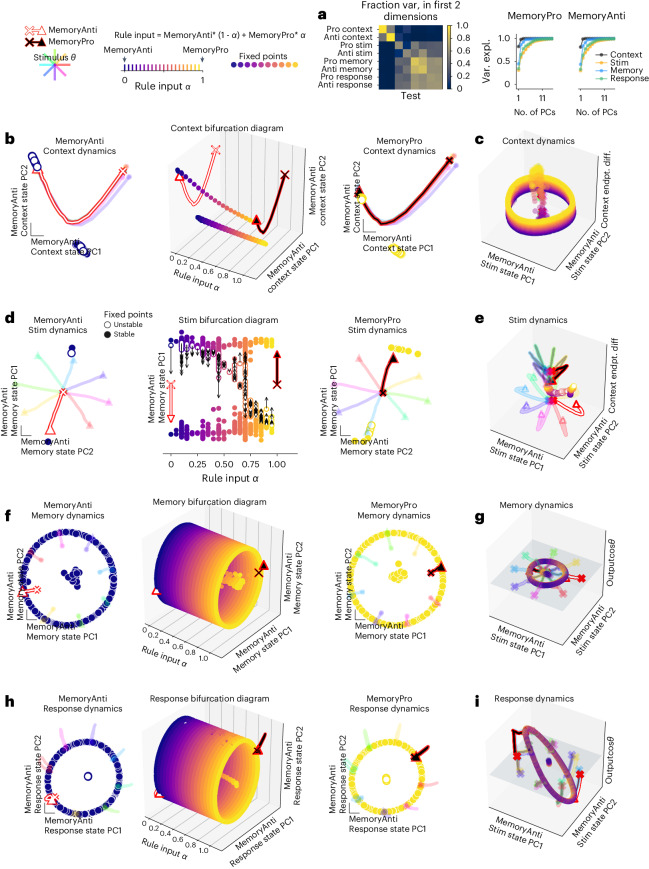
Two-task networks shared fixed points across related tasks. **a**, Fraction variance explained in each task period by top two PCs of neural state trajectories for 1,024 stimulus conditions from every other task period (left). We performed PCA on task period Y and then calculated how much variance it explained on task period X. Right: top 10 PCs of neural state trajectories for 1,024 stimulus conditions from the same task period. **b**–**i**, Fixed points for interpolation between inputs for MemoryAnti (*α* *=* 0) and MemoryPro (*α* *=* 1) tasks during context (**b**,**c**), stimulus (**d**,**e**), memory (**f**,**g**) and response (**h**,**i**) periods. **b**, Middle, fixed points for 20 intermediate *α* values (*x* axis) projected into top two PCs defined by state trajectories during the context period of the MemoryAnti task (*y* and *z* axes) with MemoryAnti *α* = 0 (left) and MemoryPro *α* = 1 (right) fixed points and trajectories. **c**, Rule input interpolation between tasks, MemoryAnti (blue fixed points, white state trajectory) and MemoryPro (yellow fixed points, black state trajectory) projected into the top two MemoryAnti stimulus period state PCs and the vector connecting context period state endpoints on the *z* axis. **d**, Same as **b** for stimulus period, with unstable (open) and stable (closed) fixed points projected into top PC defined by the state trajectories during the memory period of the MemoryAnti task (*y* axis). Local linear dynamics around unstable fixed points are shown in black arrows: the state was initialized at the unstable fixed point and run forward for 10 steps. **e**, Same as **c** for stimulus period, projected into the top two MemoryAnti stimulus period state PCs and the vector connecting context period state endpoints on the *z* axis. **f**, Same as **b** for memory period, projected into top two PCs defined by the state trajectories during the memory period of the MemoryAnti task (*y* and *z* axes). **g**, Same as **c** for memory period, projected into the top two MemoryAnti stimulus period state PCs (*x* and *y* axes) and the output weight vector (from *W*_out_) associated with cos*θ*_stimulus_ on the *z* axis. **h**, Same as **b** for response period, projected into top two PCs defined by the state trajectories during the response period of the MemoryAnti task (*y* and *z* axes). **i**, Same as **g** for response period. All subpanels were generated from the same network with hyperparameters: 256 units, softplus activation, diagonal initialization. endpt. diff., endpoint difference; Var. expl., variance explained.

**Extended Data Fig. 2:**
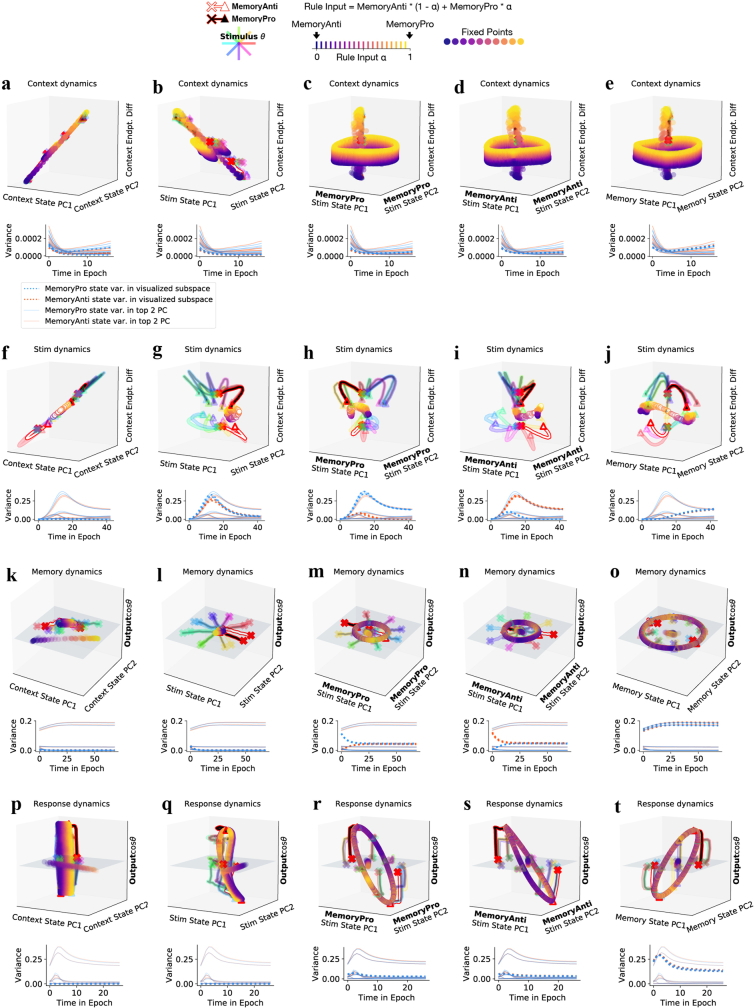
Additional visualizations of shared fixed points across MemoryPro and MemoryAnti tasks in a two-task network. These figures are meant to provide more intuition for high dimensional dynamics by presenting fixed point interpolations and state trajectories in more dimensions (**top**) with a quantification of variance of the network state across stimulus conditions plotted through time in each subspace (**bottom**). Fixed points and state trajectories for interpolation between rule inputs for MemoryAnti (*α* = 0) and MemoryPro (*α* = 1) tasks during **(a-e)** context **(f-j)** stimulus **(k-o)** memory **(p-t)** and response periods. **(a-e) top**: Context period fixed points for rule input-interpolation between tasks, MemoryAnti (blue fixed points, white state trajectory) and MemoryPro (yellow fixed points, black state trajectory) projected into the top two principal components (PCs) identified by performing PCA on concatenated state trajectories from MemoryPro and MemoryAnti **(a)** context task periods **(b)** stimulus task periods **(c)** MemoryPro stimulus task period only **(d)** MemoryAnti stimulus task period only **(e)** memory task periods. **(a-e) bottom**: Variance of state trajectories across stimulus conditions plotted through time during MemoryPro (blue, dotted) and MemoryAnti (orange, dotted) projected in the associated (above) visualized subspace. Solids lines indicate variance of state trajectories during the context period, projected into their own top two PC subspace. This provides a reference for how much variance the visualization captures relative to the PC subspace for each task period. Note, there is little variance during the context period, especially in networks only trained to perform a small number of tasks. Therefore, visualizations in the leftmost column and top row do not provide much insight about the dynamics, but more clearly show the relationship between the stable fixed point that guides the state during the context period and the ring attractor during the memory period, both shared across tasks. Remember, inputs during context and memory periods are identical; and therefore, fixed points are identical across these task periods. **(f-j)** Same as **a-e** for stimulus period fixed points and state trajectories. These panels show that the (**g)** initial stimulus response is in an overlapping subspace; however, (**h,i)** there are mostly nonoverlapping subspaces that capture maximal stimulus variance across both tasks. **(j)** By the end of the stimulus period, the state moves into an aligned memory subspace across both tasks. **(k-o)** Same as **a-e** for memory period fixed points and activity. **(l)** Early stimulus period representation is mostly orthogonal to memory period representation. **(m-n)** State trajectories move out of separate stimulus subspaces early in the memory period. **(o)** Little movement of state trajectories in shared memory subspace throughout memory period. **(p-t)** Same as **a-e** for response period fixed points and state trajectories show **(r-t)** similar organization of fixed point and trajectories in all subspaces. All subpanels generated from the same network as in Fig. 2 with hyperparameters: 256 units, *softplus* activation, diagonal initialization.

We then trained networks to perform two tasks on interleaved batches. The MemoryPro and MemoryAnti tasks were both memory-guided response tasks that received identical stimulus inputs. The target outputs in the pro task were the same as the stimulus inputs (*φ*_response_ *=* *θ*_stimulus_), whereas, in the anti task, targets were in the opposite direction as the stimulus (*φ*_response_ *=* *θ*_stimulus_ + π) (see Extended Data Fig. 1 and 'Tasks and performances' in Methods for full task definitions). We quantified the fraction of state trajectory variance explained in the top 10 PCs for a given task period and in the top two PCs defined by every other task period (Fig. 2a). This provided a reference for the fraction of variance captured in visualizations (Fig. 2b–i). We visualized and quantified variance of the network state in each task period for many additional subspaces (Extended Data Fig. 2).

**Extended Data Fig. 3:**
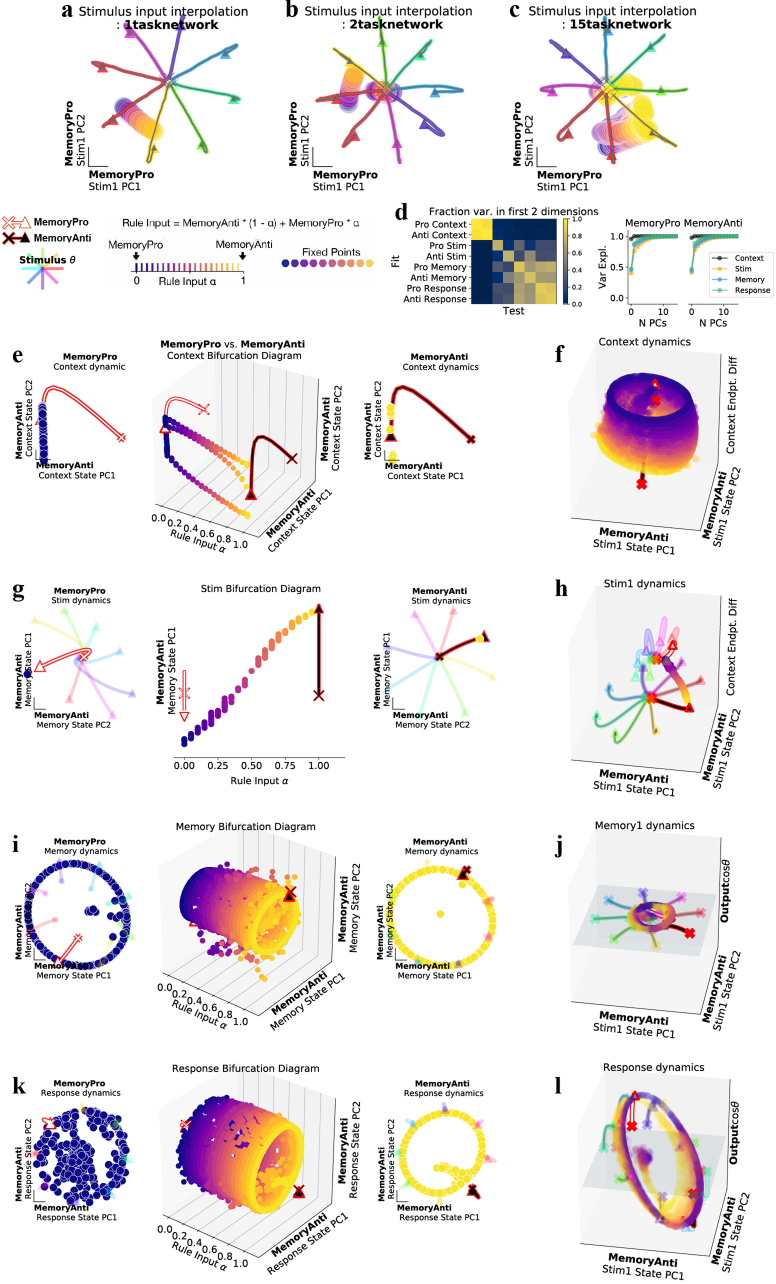
Fixed point structure is dependent on other tasks the network is trained to perform and fixed points are shared regardless of network size or noise. **(a-c)** Networks were trained to perform 1, 2 or 15 tasks with the same hyperparameters: LeakyRNN, 256 units, *softplus* activation, diagonal initialization. **(a)** One-task network fixed points for interpolated inputs across stimulus conditions 0°(blue)→45° (yellow), reveals one fixed point that moves dependent on stimulus input conditions. Trajectories colored according to stimulus orientation. Additional fixed points are revealed in **(b)** two task network and **(c)** 15 task network. Subpanels **a-c** reveal that training a network on multiple tasks results in additional fixed points that persist across different contextual inputs. This highlights a key point, that neural dynamics in real brains will likely be a product of all the tasks animals perform. This result could guide future perturbation experiments and the study of error trials. Rotations of fixed points in the PC spaces of different networks is irrelevant to this point. **(d-l**) Large networks with no noise also share fixed points. Example two task network trained to perform MemoryPro and MemoryAnti tasks with hyperparameters: LeakyRNN, *1024* units, *softplus* activation, diagonal initialization and no input noise or independent noise (see equations 5 and 10 in Methods for noise definitions). (**d**) Fraction variance explained in each task period by **left:** top two principal components (PCs) of state trajectories for 1024 stimulus conditions from every other task period. We performed PCA on task period Y then calculated how much variance it explained on task period X. **right:** top ten PCs of state trajectories for 1024 stimulus conditions from the each task period. **(e-l**) Fixed points for interpolation between inputs for MemoryPro (*α* = *0*) and MemoryAnti (*α* = *1*) tasks during **(e,f**) context **(g,h**) stimulus **(i,j**) memory **(k,l**) and response periods. **(e**) **middle:** Fixed points for 20 intermediate *α* values (x-axis) projected into top two PCs defined by state trajectories during the context period of the MemoryAnti task (y and z-axis) with (**left)** MemoryPro *α* = *0* and **(right)** MemoryAnti *α* = *1* fixed points and trajectories. **(f**) Fixed points for rule input-interpolation between tasks, MemoryPro (blue fixed points, white state trajectory) and MemoryAnti (yellow fixed points, black state trajectory) projected into the top two MemoryAnti stimulus period state PCs and the dimension separating both tasks at the end of the context period (z axis). **(g**) Same as **e** for stimulus period, projected into top PC defined by state trajectories during the memory period of the MemoryAnti task (y-axis) **(h**) Same as **f** for stimulus period. **(i**) Same as **e** for memory period, projected into top two PCs defined by state trajectories during the memory period of the MemoryAnti task (y and z-axes). **(j**) Same as **h** for memory period, projected into the top two MemoryAnti stimulus period state PCs (x and y-axes) and the output weight vector (from *W*_*out*_) associated with *cosθ*_*stimulus*_ on the z-axis. **(k**) Same as **e** for response period, projected into top two PCs defined by the state trajectories during the response period of the MemoryAnti task (y and z-axes). **(l**) Same as **j** for response period.

Input interpolation across rule inputs for a network trained on the MemoryPro and MemoryAnti tasks revealed shared fixed points across tasks during the context/memory, stimulus and response periods (Fig. 2). Context period fixed points were similar to the single-task network throughout rule input interpolation, with one stable fixed point that was relevant to the context period and a ring of fixed points that was relevant to the memory period (Fig. 2b,c,f,g). Stimulus period rule input interpolation revealed two separate stable fixed points and an unstable fixed point between them for each intermediate input condition (Fig. 2d,e). The network state evolved away from the unstable fixed point, which smoothly moved in state space across interpolated input conditions, resulting in the network state evolving toward a different stable fixed point for each task. From that point onward, the state interacted with a shared ring attractor across both tasks (MemoryPro and MemoryAnti) and task periods (memory and response) according to the response direction (Fig. 2f–i). In summary, this network flexibly performed two related computations through small changes in fixed point locations. In addition to shared fixed points across different tasks and task periods, we could identify shared fixed points across different stimulus conditions for the same task period (Extended Data Fig. 3a–c).

One might expect that networks share fixed points due to the limited computational resources in small networks. We, therefore, trained networks that were nearly an order of magnitude larger and without noise in the inputs or recurrent units to determine whether supplying abundant computational resources might change this solution. To our surprise, we found that even large networks without noise still shared dynamical motifs (Extended Data Fig. 3d–l). We interpret these findings to mean that shared dynamical motifs are not a product of limited resources, and we explore possible explanations for shared motifs in 'Discussion'. Examples in Fig. 2 and Extended Data Fig. 3d–l provide a demonstration of what is and is not consistent across different networks. It is consistent that fixed points persist across different inputs and are often shared across tasks. On the other hand, the configuration of fixed points is not consistent across contexts (Fig. 2d and Extended Data Fig. 3g).

### Identifying dynamical motifs in 15 task networks

To quantify shared structure across many tasks in a single network and to compare shared structure across multiple networks, we developed a modified version of the task variance metric described by Yang et al.^21^. We were motivated to study task periods because changes in the inputs reconfigure the RNN’s dynamics across task periods. For example, when the stimulus input turns off in some tasks, the network goes from processing a stimulus to maintaining a memory of the stimulus. Task periods, therefore, provide the relevant granularity to identify the dynamical motifs that perform distinct computations.

**Fig. 3:**
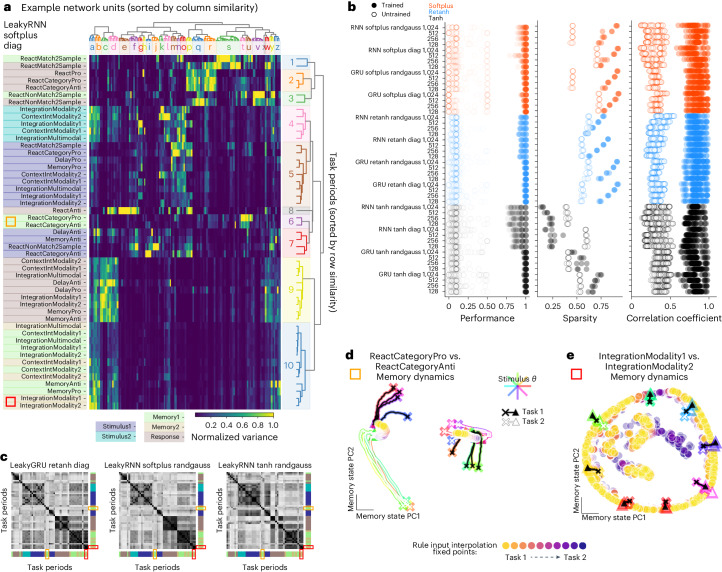
Modular organization in 15 task networks was not dependent on activation function, network initialization or size. **a**, Variance matrix: variance of unit activations across stimulus conditions, normalized across task periods (columns normalized by the maximum entry in each column). Rows and columns were sorted according to similarity ('Clusters' in Methods). Colors along the left side label each task period type (dark blue: initial stimulus; light blue: second stimulus; light green: initial memory; yellow: second memory; brown: response). Notice that blocks with high variance belong to same task period types (similar colors). **a**,**c**–**e**, Orange and red rectangles highlight category memory (ReactCategoryPro, ReactCategoryAnti) and continuous memory (IntegrationModality1, IntegrationModality2) tasks, respectively. **b**, Left: performance across all tasks for three networks of each of 48 hyperparameter settings. Middle: sparsity of the task variance measured as the fraction of entries <15% maximum unit variance. Right: task period correlation matrix (examples shown in **c**) for trained and untrained networks are sorted according to rows in **a** and correlated to trained networks for all other hyperparameter settings. **c**, Correlation matrix of rows in variance matrix (as in **a**) for three different example networks; rows and columns sorted according to rows in **a**. Colors along the bottom and right axes label each task period type (dark blue: initial stimulus; light blue: second stimulus; light green: initial memory; yellow: second memory; brown: response). Notice that blocks with high correlation belong to the same task period types (similar colors). **d**, Shared point attractors for two category memory tasks as seen by input interpolation across tasks during memory period. State trajectories for eight stimulus conditions (colored by stimulus direction) starting from ‘x’ projected in PC space, defined by concatenating memory period state trajectories from both tasks for ReactCategoryPro (black) and ReactCategoryAnti (white) tasks. Rule input interpolation across tasks during memory period with fixed points for intermediate rule input conditions in filled circles. **e**, Same as **d** for two continuous circular variable memory tasks, highlighting shared ring attractors. State trajectories starting from ‘x’ projected in PC space, defined by concatenating memory period state trajectories from both tasks for IntegrationModality1 (black) and IntegrationModality2 (white) tasks. Panels **a**, **d** and **e** were generated from the same network with hyperparameters: 128 units, softplus activation, diagonal initialization. randgauss, random Gaussian.

**Extended Data Fig. 4:**
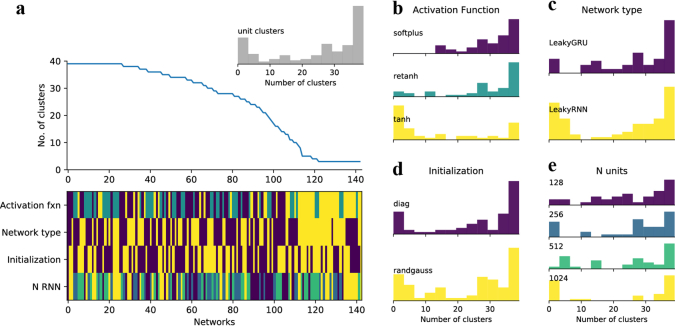
Activation function dictates whether clusters emerge in a network. A total of 144 networks were trained, each with different hyperparameters. **(a) top:** Networks are sorted by their numbers of clusters. **bottom**: the hyperparameters used for each network are indicated by colors, as defined in **b-e**. Inset, the distribution of cluster numbers across all networks. **(b-e)** Breaking down the number of clusters according to the **(b)** activation function **(c)** network architecture **(d)** weight initialization **(e)** number of units. Only networks that reached the minimum performance of 80% for every task are shown.

We divided tasks into task periods and computed the variance across stimulus conditions for each unit, normalized across all task periods ('Task variance analysis' in Methods). The result was a matrix of each unit’s normalized variance for each task period of every task (Fig. 3a), which we refer to as the variance matrix in subsequent analyses. We sorted the rows and columns of this matrix based on similarity ('Clusters' in Methods). Clusters of units a–z were identified by performing hierarchical clustering on the columns and rows of the variance matrix and identifying a distance criterion to maximize the ratio of intercluster to intracluster distances (Fig. 3a, Extended Data Fig. 4 and 'Clusters' in Methods).

**Extended Data Fig. 5:**
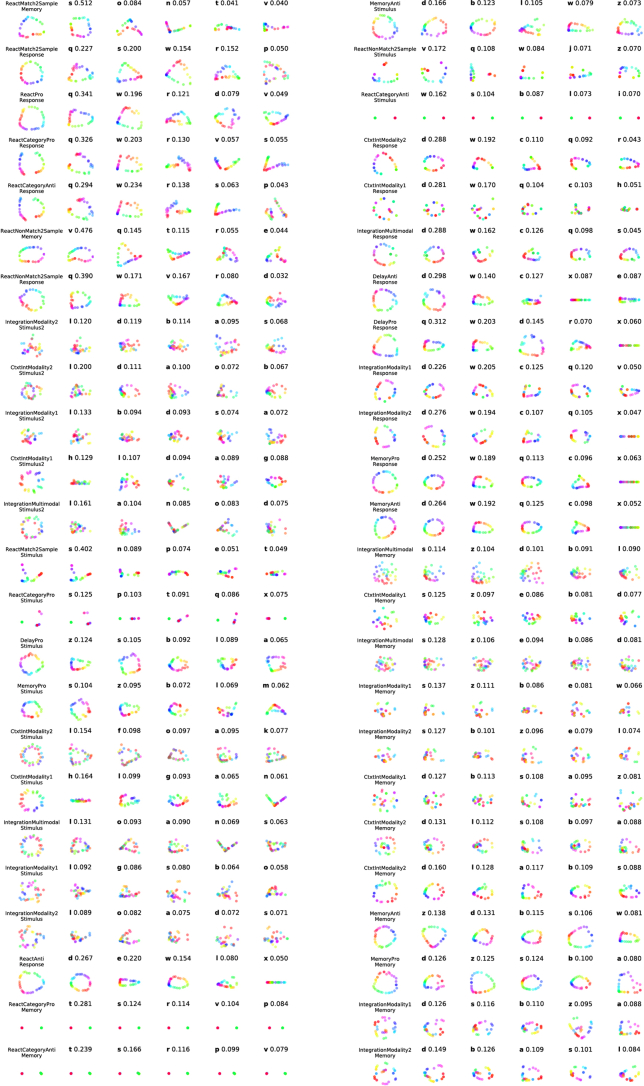
Qualitative structure and quantitative variance of the fixed points in each unit cluster. For each task period, we identified the fixed point most proximal to the network state at the end of the task period for thirty-two different input conditions. Fixed points most proximal to the network state were selected to focus our analysis on the most relevant fixed points for each task period. This analysis resulted in identification of thirty-two relevant fixed points for each task period that span stimulus conditions. We visualized the projection of these thirty-two fixed points in the top 2 PCs of the activity of all units (column 1) and in the top 2 PCs each unit cluster’s activity (column 2–6, letters denote unit cluster identity as in Fig. 3a). Due to space constraints, we visualize qualitative projections and quantification of variance for only the top 5 unit clusters, which capture maximal variance for each task period. In general, the rest of the unit clusters not shown captured less than 10% of the total variance each. For clusters with only one unit, we visualized this projection in one dimension (cluster x). Unit cluster identification (letters a-z) and normalized variance across fixed points projected into the space of all units within the cluster indicated above each visualization. We normalized variance by the sum of variance across all clusters. Rows are presented in the same order as the variance matrix for comparison (Fig. 3a).

**Extended Data Fig. 6:**
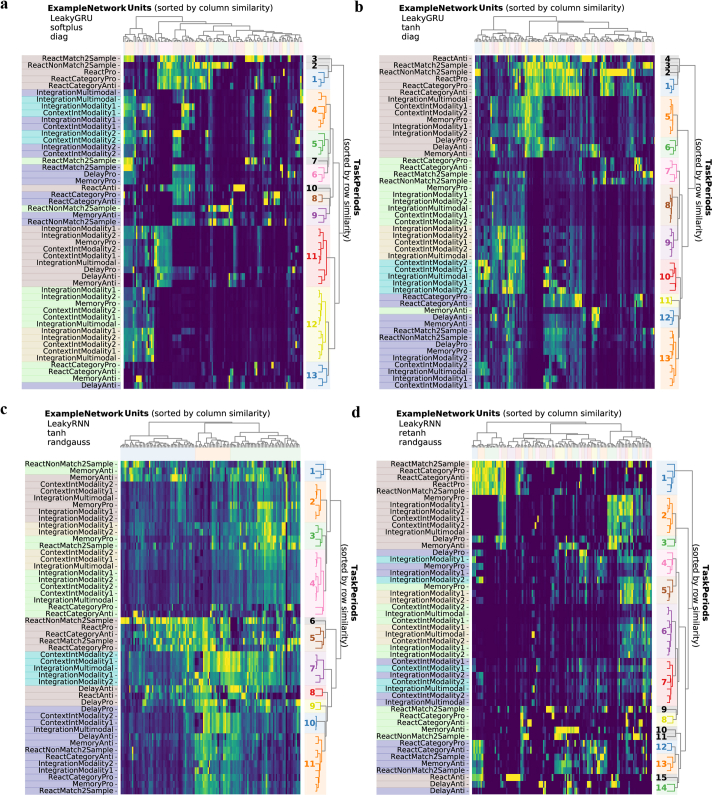
Example variance matrices for four different networks. Variance matrix for four example networks. Variance of unit activations across stimulus conditions, normalized across task periods (columns normalized by the maximum entry in each column). Rows and columns sorted according to similarity. Colors along left side label each task period type (dark blue: initial stimulus, light blue: second stimulus, light green: initial memory, yellow: second memory, brown: response). Notice blocks with high variance belong to same task period types (similar colors). Colors along the right side highlight task periods that were grouped together in clusters according to the optimal distance threshold for each dendrogram by computing the silhouette score based on intracluster and intercluster distances (see Clusters in Methods). **(a)** LeakyGRU, *softplus*, diagonal initialization, *N*_rec_ = 128 **(b)** LeakyGRU, *tanh*, diagonal initialization, *N*_rec_ = 128 **(c)** LeakyRNN, *tanh*, random Gaussian initialization, *N*_rec_ = 128 **(d)** LeakyRNN, *retanh*, random Gaussian initialization, *N*_rec_ = 128.

Sorting the rows and columns of the variance matrix revealed a blockwise structure, where groups of units had large variance for groups of task periods with similar computational requirements (Fig. 3a). Similar computations can be seen in the task period color labels (Stimulus1 and Stimulus2—for tasks with two sequential stimulus presentations, Memory and Response) and in the task names (Category, DecisionMaking, Memory, and so on) (Fig. 3a, left). For example, task period cluster 2 (Fig. 3a, right) corresponds to reaction-timed response task periods (see Extended Data Fig. 1 for definitions of all tasks). These tasks receive new stimulus information during a response period that must be incorporated into the computation immediately. Therefore, the network cannot prepare a response direction before the fixation cue disappears. On the other hand, in task period cluster 9, the network receives no new information during the response period and must, instead, use the memory of the stimulus to produce the correct output during the response period. These separate blocks in the variance matrix reveal two distinct clusters of units that contribute to response period dynamics: one for tasks with reaction-timed responses and another for tasks with memory-guided responses. Other unit clusters for stimulus (unit clusters k–o, task period clusters 4 and 5) and memory (unit clusters a and b, task period cluster 10) computations are apparent in the block-like structure aligned with task period type (Fig. 3a, task titles and task period color labels to the left of the variance matrix). Qualitative structure and quantitative variance of the fixed points for each task period within unit clusters demonstrate the relationship between dynamical motifs and unit clusters (Extended Data Fig. 5). Block structure in the variance matrix was robust to different network architectures and hyperparameters (examples in Extended Data Fig. 6). To quantify task period similarity across networks with different hyperparameters, we calculated the variance matrix for each trained network. We then sorted task periods according to similarity of rows in one reference network and computed the correlation matrix of the sorted rows in the variance matrix for each network ('Task variance analysis' in Methods). The correlation matrix for each trained network revealed the block-wise similarity of task periods (Fig. 3c). Higher correlation across trained networks compared to untrained networks confirmed that the block structure in the variance matrix emerged from learning the task computations rather than from network design choices or the structure of the inputs (Fig. 3b, right) (Pearson correlation coefficient between correlation matrices; 'Task variance analysis' in Methods).

**Extended Data Fig. 7:**
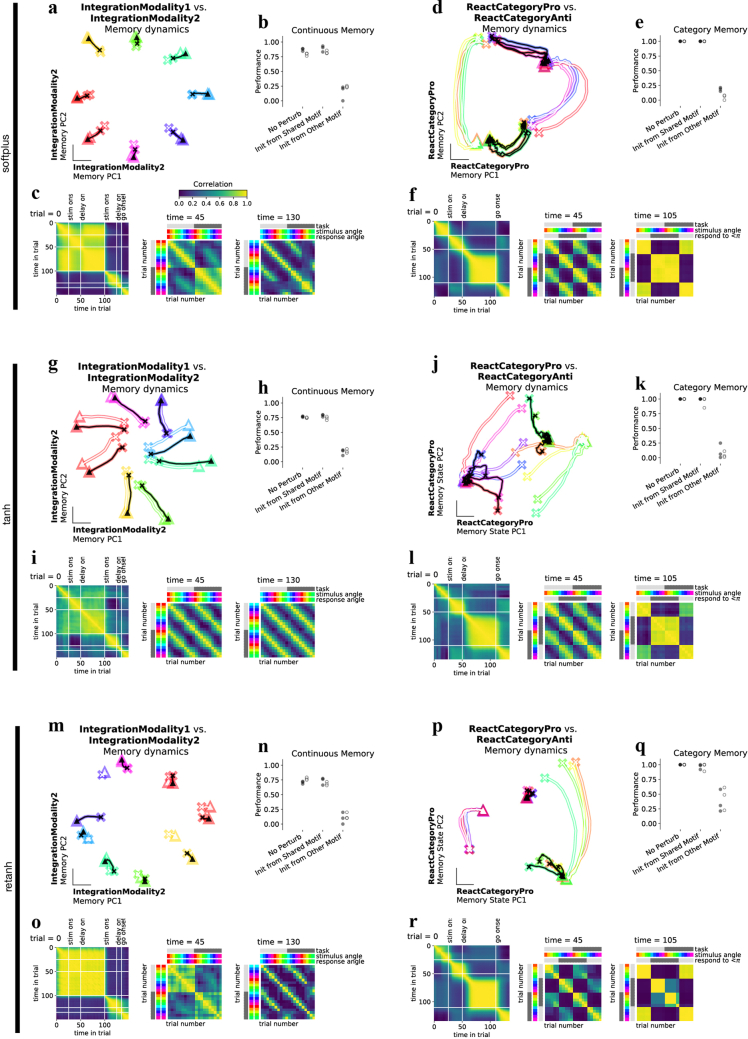
Discrete and continuous variable memory computations result in two kinds of shared memory attractors. We utilize analyses that could be performed in biological neural data or through behavioral experiments to highlight shared category and continuous memory dynamical motifs in networks with different activation functions. Networks were trained with *softplus* activation function for **a-f (a)** Neural state trajectories for eight stimulus conditions (colored by response direction) starting from ‘x’ in IntegrationModality2 PC space for IntegrationModality1 (white) and IntegrationModality2 (black) tasks. **(b)** Perturb initial condition for IntegrationModality1 response period state to the memory period final state of either IntegrationModality2 task (Init from shared motif), ReactCategoryAnti (Init from other motif) or IntegrationModality1 (No perturb) for trials with the same response direction and run the network forward through the response period. One data point for each of three networks trained with diagonal initialization (filled circles) or with a random Gaussian initialization (open circles). Performance was similar across ‘No perturb’ and ‘Init from shared motif’ conditions, but was dramatically impaired for ‘Init from other motif’ conditions. **(c) left**: Correlation across timesteps throughout an IntegrationModality1 trial. Task period transitions highlighted in white lines. **middle**: Correlation across trials for different stimulus conditions for IntegrationModality1 and IntegrationModality2 tasks at the end of the first stimulus period. right: Correlation across trials for different stimulus conditions for IntegrationModality1 and IntegrationModality2 tasks at the end of the memory period. Trials are highly correlated across tasks according to response direction. **(d)** Same as **a** for two category memory tasks, ReactCategoryPro (white) and ReactCategoryAnti (black). **(e)** Perturb initial condition for ReactCategoryPro response period state to the memory period final state of ReactCategoryAnti task (Init from shared motif), IntegrationModality1 (Init from other motif) or ReactCategoryPro (No perturb) for trials with the same response direction and run the network forward through the response period. **(f) left**: Correlation across timesteps throughout an ReactCategoryPro trial. Task period transitions highlighted in white lines. middle: Correlation across trials for different stimulus conditions for ReactCategoryPro and ReactCategoryAnti tasks at the end of the stimulus period. **right**: Correlation across trials for different stimulus conditions for ReactCategoryPro and ReactCategoryAnti tasks at the end of the memory period. Trials are highly correlated across tasks according to response direction. **(g-l)** Same as **a-f** for *tanh* activation function. **(m-r)** Same as **a-f** for *retanh* activation function. One example network in panels **a,c,d,f** (*softplus* activation), **g,i,j,l** (*tanh* activation) and **m,o,p,r** (*retanh* activation) where all three example networks have hyperparameters: LeakyRNN, 128 units, diagonal initialization.

The variance matrix quantified the extent to which the neural state evolved in similar subspaces across task periods, suggestive of which computations shared dynamical motifs. We highlight two different examples of shared memory dynamical motifs using rule input interpolation (Fig. 3d,e) and highlight their positions in the variance matrix (Fig. 3a, left of task period names, red and yellow squares). A pair of category memory task periods are within the same cluster in the variance matrix, suggesting that their computations are performed by a similar set of units (Fig. 3a, yellow square left: task period cluster 6, unit clusters t and u). Both category tasks used the same two point attractors for memory of the initial stimulus. Rather than store the identity of the initial continuous circular stimulus, the network stored which category it must respond to, regardless of task (Fig. 3d). In another example of a shared attractor across tasks, we found a ring attractor that was shared across several tasks (Fig. 3a; task period clusters 9 and 10, unit clusters a–d). All these tasks required memory of the initial continuous circular stimulus variable. To show that this ring attractor was shared across tasks, we interpolated across rule inputs for a pair of these tasks (IntegrationModality1 and IntegrationModality2) and found a similar shared ring structure as in the two task networks (Fig. 2f,g). We highlight shared category and continuous memory dynamical motifs in networks with different activation functions in Extended Data Fig. 7.

In addition to clusters of task periods with similar variance, there were also some task periods that did not cluster with other task periods. For example, task period cluster 8 is dedicated to the ReactAnti task; cluster 1 is dedicated to the ReactMatch2Sample task; and cluster 3 is ReactNonMatch2Sample (Fig. 3a). In these cases, the computation performed in the unique task period is so distinct from other computations, the dynamical motif is unlikely to be reused across tasks. These results are robust; the set of tasks that employed unique dynamical motifs was similar across hyperparameter settings (Fig. 3c and Extended Data Fig. 6).

### Motif alignment to unit axes

**Extended Data Fig. 8:**
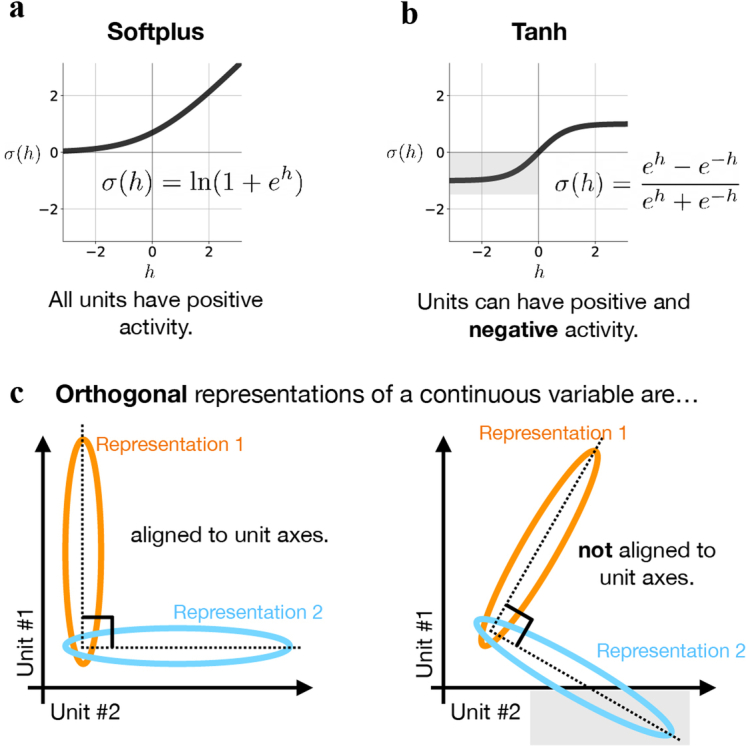
Positive activation functions align orthogonal motifs to unit axes. **(a)** *softplus* activation function maps activity to the positive real line. **(b)** *tanh* activation function does not impose positivity constraints. **(c) left**: Two activity patterns that are constrained to lie in the positive orthant can only be orthogonal to one another when aligned to the unit axes or with elevated activity levels (which is penalized as a biological constraint). **right:** Activation functions that result in positive or negative activity do not constrain activity patterns to unit axes. *Note: Either system can use additional dimensions to orthogonalize representations not aligned to unit axes in an infinitely large network*.

One notable difference across network hyperparameters was sparsity in the variance matrix. We define sparsity to be the fraction of entries in the variance matrix below a threshold of 15% maximum unit variance. Networks with non-negative activation functions had sparse task variance matrices, whereas networks with the tanh activation function, which has a range of (−1, 1), did not (Fig. 3b, middle). We understand sparsity to be a function of optimal network performance requiring potentially interfering dynamical motifs to be organized into orthogonal subspaces. In a network with activity regularization and where all units can take only positive values, this orthogonalization favors unit axes (Extended Data Fig. 8). We found clusters to be present in tanh networks, simply not aligned to unit axes and, therefore, non-identifiable using methods described in Yang et al.^21^. By examining the correlation matrix and the correlation coefficient across networks, we see that similar clusters are present in the tanh networks (Fig. 3b, right, and Fig. 3c, right).

### Shared stimulus period dynamical motifs in 15 task networks

The variance matrix provides a useful overview of which task periods are implemented by similar clusters of units but falls short of addressing exactly how these subpopulations implement shared dynamical motifs. Shared motifs are implemented by organizing the state in the appropriate region of state space to evolve on the relevant shared dynamical landscape. To walk through this explanation in detail, we focus on stimulus period dynamics and highlight two examples, one in which dynamical motifs are shared and another where motifs are not shared.

**Fig. 4:**
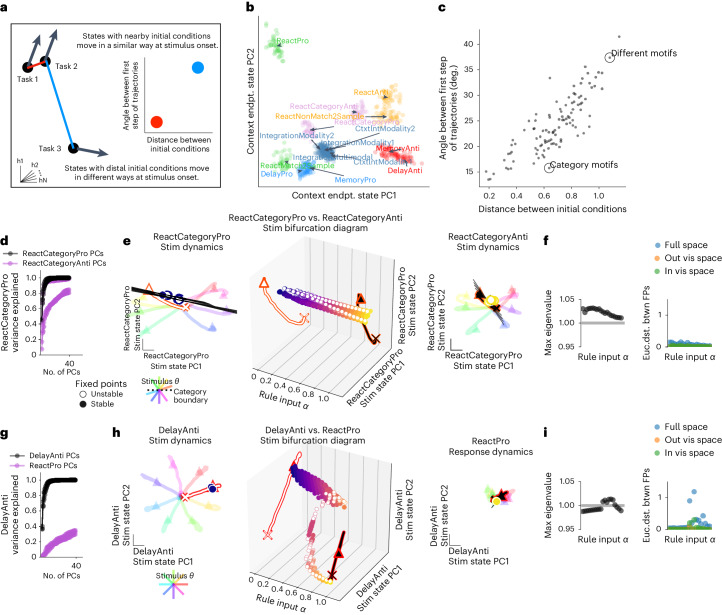
Tasks with similar stimulus computations were in nearby parts of state space and shared dynamical motifs. **a**, Schematic of analyses in **b** and **c**. **b**, The state for each trial (colored dot for each of 20 trials on each task) at the end of the context period (just before stimulus period) projected onto the top two PCs defined by the state at the end of the context period for all tasks. Trial–trial variation is due to input noise, independent noise and random initial conditions. Trials colored by similar stimulus computations as given by task definitions: pro delayed (light blue), anti delayed (red), pro integration (steel blue), categorization (pink), pro reaction (green) and anti reaction (orange). See Supplementary Table 1 for motif definitions. **c**, Euclidean distance between pairs of trials from different tasks at the end of the context period plotted against cosine angle between same pair after stimulus onset for a particular stimulus input, *θ*_stimulus_, for one timestep and then averaged across stimulus angle inputs. Pairs of tasks in **d**–**i** are circled and labeled: ‘Category Motif’: ReactCategoryPro and ReactCategoryAnti (**d**–**f**) and ‘Different Motifs’: DelayAnti and ReactPro (**g**–**i**). **d**, Fraction of variance explained for ReactCategoryPro task by the ReactCategoryAnti task PCs (purple) compared to its own PCs (black) for five trained networks with different random seeds. **e**, Rule input interpolation across category tasks for one stimulus angle. Middle, unstable (open) and stable (closed) fixed points for 20 intermediate *α* values (*x* axis) projected onto top two PCs defined by state trajectories during the stimulus period of the ReactCategoryPro task (*y* and *z* axes) with ReactCategoryPro *α* = 0 (left) and ReactCategoryAnti *α* = 1 (right) fixed points and trajectories for eight different stimulus angles (rainbow colors). Expanding dimensions around unstable fixed points are visualized as black lines. **f**, Left, maximum real eigenvalue for the linearization of the state update around each fixed point for the single unstable fixed point closest to the state at the end of the stimulus period for 20 consecutive *α* values between 0 and 1. Right, Euclidean distance between consecutive fixed points in the full state space (blue), outside of the visualized subspace in **e** (orange) and within the visualized space (green). We analyzed only one unstable fixed point that is most proximal to the end of the state trajectory for each input condition to highlight the task relevant fixed point ('Analysis of fixed points for interpolated inputs' in Methods). **g**–**i**, ‘Different Motifs’: DelayAnti and ReactPro. Same as **d**–**f** but for DelayAnti and ReactPro tasks. Note that a response is required at stimulus onset for the ReactPro task; therefore, the stimulus period is the same as the response period. **i**, Left, maximum real eigenvalue for the linearization of the state update around each fixed point for the single fixed point closest to the state at the end of the stimulus period for 20 consecutive *α* values between 0 and 1. Right, Euclidean distance between consecutive fixed points in the full state space (blue), outside of the visualized subspace in **h** (orange) and within the visualized space (green). We analyzed one fixed point that is most proximal to the end of the state trajectory for each input condition. Panels **b**, **c**, **e**, **f**, **h** and **i** were generated from the same network as in Fig. 3a with hyperparameters: LeakyRNN, 128 units, softplus activation, diagonal initialization. Other networks in **d** and **g** use the same hyperparameters. deg, degrees; endpt., endpoint; Euc.dist. btwn, Euclidian distance between; FP, fixed point; vis, visualized.

**Extended Data Fig. 9:**
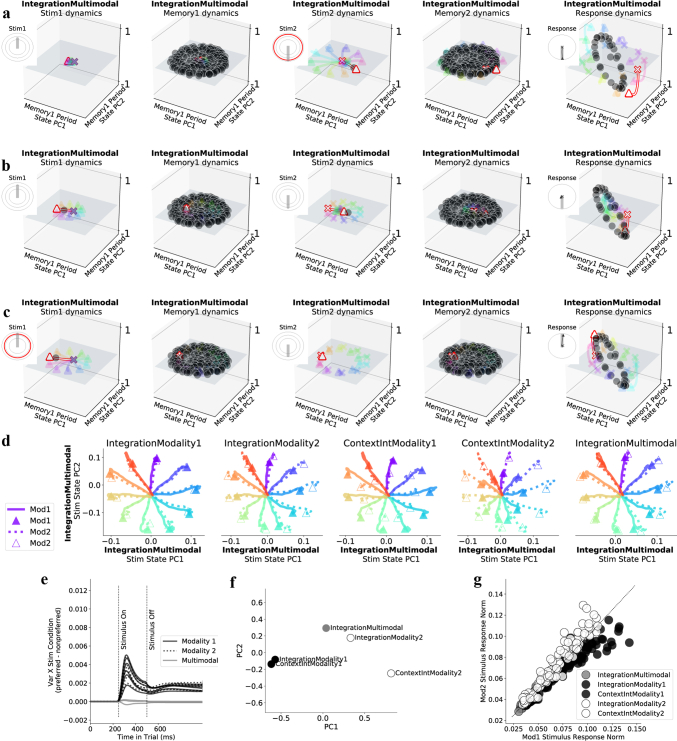
Integration tasks implement memory on a plane attractor; with stimulus processing through fixed points and contextual input amplification. Here we present fixed point structures and context dependent input responses for integration of a noisy continuous variable. **(a-c)** Fixed points and state trajectories during the multimodal integration task in one example network trained to perform all fifteen tasks. **(a)** All panels are different task periods from one trial where the first stimulus has a small amplitude, pointing upward and the second stimulus has a large amplitude pointing downward in both modality1 and modality2. Fixed points and state trajectories projected into PC space defined by state trajectories during the memory period of the IntegrationMultimodal task. Insets show stimulus direction and amplitude for both modalities: small amplitude stimuli reach the inner circle and large amplitude stimuli reach the outer circle. Large amplitude stimulus periods highlighted in red. From left to right panels: small amplitude input during first stimulus presentation produces dynamics that lack stable fixed point, resulting in the state remaining near initial condition. Memory period inputs result in a plane of marginally stable fixed points that maintain the position of the state after stimulus period 1. Stimulus period 2 has a large amplitude stimulus input, resulting in a single stable fixed point that attracts the state. The second memory period has the same inputs as the first memory period and therefore results in the same plane of marginally stable fixed points that maintain the position of the state after stimulus period 2. The response period inputs result in a ring of fixed points in output potent space that attracts the state to a region on the ring, dependent on initial conditions at the beginning of the response period (end of the memory period). Inset shows target output direction in thick gray line and actual outputs in thin black line emanating from ‘x’ and ending with ‘▴.’ Network correctly responds in the downward direction. **(b)** Same as **a** during one trial where first and second stimuli have medium amplitude. Identical amplitude inputs mean correct response direction is chosen at random. Network responds weakly downward. **(c)** Same as **a** during one trial where first stimulus has a large amplitude, pointing upward and the second stimulus has a small amplitude pointing downward. Network correctly responds upward. These panels demonstrate how fixed points guide the network to perform an integration task. **(d**) Demonstration of contextual input amplification for context dependent integration tasks. Stimulus dependent network state for both modality 1 (solid) and 2 (dotted) for five different tasks where relevant modality is specified in title for each subpanel. During IntegrationModality1 and IntegrationModality2 tasks, only the relevant stimulus is presented, but here we present either to compare state trajectories for each stimulus condition across tasks. Notice for contextual integration tasks, the state moves a greater distance from the origin in response to the relevant modality; whereas in the multimodal task, responses are fairly equal. **(e**) Quantification of contextual differences in **d**: Difference between variance across stimulus conditions for preferred modality minus variance across stimulus conditions for nonpreferred modality. Preference is defined by task rules. One line for each of five tasks in three different networks with different random seeds. **(f**) Context period network state for all five tasks projected onto the first and second PCs defined by variance of the state across tasks at the end of the context period (PCA on 5 × *N*_rec_ matrix). Organization of network states at the end of the context period shows compositional structure. PC1 goes from modality 1- > 2 and PC2 separates ContextIntegration vs. Integration decision tasks. **(g)** Norm of input response for twenty-four stimulus conditions spanning [0, 2π) on each of five tasks (see Effective input modulation Methods). Trials where the network should prefer Modality 1 are black, Modality 2 white, multimodal gray. It will be an interesting direction for future work to determine whether other features of the dynamics, such as input response amplification, could be shared across contexts in a similar manner to how fixed points are shared. This could be done through interpolation of initial conditions with identical inputs. Hyperparameters: LeakyRNN, *softplus* activation, diagonal initialization, *N*_rec_ = 128.

Tasks with similar stimulus computations (integration, categorization, pro versus anti and reaction-timed versus delayed response) organized stimulus period initial conditions to be nearby in state space and evolve in a similar way after stimulus onset (see schematic in Fig. 4a). We visualized this organization in PC space defined by the final state of the context period across all tasks (Fig. 4b). To summarize the relationship between initial conditions and the ensuing stimulus dynamics for different tasks, we compared pairs of trials presented with the same stimulus across different tasks. We plotted the Euclidean distance between initial conditions against the angle between the state vector on the first timestep for pairs of trials (Fig. 4c). We observed that pairs of tasks with similar computations had initial conditions that were closer together and had smaller angles between state trajectories on the first timestep of the stimulus period compared to pairs of tasks with distinct computations. Similar initial conditions for stimulus onset resulted in shared context-dependent stimulus amplification in some networks (Extended Data Fig. 9). In these cases, the state update was scaled in magnitude according to whether the stimulus input was either Modality 1 or Modality 2, dependent on the position of that state at stimulus onset^30^.

The relationship between context period states and stimulus period trajectory angles across tasks supports the idea that nearby initial conditions allowed tasks with similar stimulus computations to reuse the same dynamical landscape and, therefore, evolve in similar ways. We examine these features in two examples of comparisons between tasks that (1) share the same stimulus period dynamical motif and, then, tasks that (2) do not share the same dynamical motif (Fig. 4d–i).

In the case of two categorization tasks, ReactCategoryPro and ReactCategoryAnti, we found a shared stimulus motif (Fig. 4d–f). In the ReactCategoryPro task, the network was trained to respond if both sequential stimuli were less than or both greater than π; whereas, in the ReactCategoryAnti task, the network was trained to respond if stimuli were on opposite sides of π. In either task, there was a decision boundary at *θ*_stimulus_ = π. The initial conditions for these tasks were nearby, and trajectories during the stimulus period were aligned (Fig. 4c, ‘Category Motif’). We quantified stimulus response overlap by computing the fraction of variance explained for the state trajectory during one task by the other task’s PCs (purple) compared to its own PCs (black) (Fig. 4d), revealing that both tasks were performed in an aligned subspace. Aligned stimulus responses for both category tasks were visualized in PC space defined by the stimulus period state trajectories of the ReactCategoryPro task (Fig. 4e, left and right). These analyses revealed that activity evolved in a qualitatively similar way for trials with the same stimulus conditions during both tasks, suggesting that the state trajectories could have occurred on a similar dynamical landscape.

To better understand the relationship between the dynamical landscapes across task contexts, we interpolated across rule inputs during the stimulus period for both category tasks with the same stimulus input. We found that similar stimulus responses were governed by shared stable and unstable fixed points, demonstrated by the smooth bridge of fixed points between both tasks (Fig. 4e, middle). We projected the unstable dimension of each unstable fixed point into PC space and found that this dimension was aligned with the direction of the state trajectories and roughly orthogonal to the decision boundary. (Fig. 4e). We defined the most relevant fixed point to be the closest unstable fixed point to the state at the end of this task period. This simplification of one relevant fixed point was often necessary to tease apart how relevant dynamics are reconfigured across tasks while several to hundreds of other fixed points related to computations during other task periods also moved through state space. A continuous bridge mapped movement of the relevant fixed point, suggesting that rule inputs shifted a relevant shared fixed point that was reused across both tasks (Fig. 4e,f). Moreover, the stability of the local linear dynamics around this shared fixed point was consistent across all intermediate input conditions, as shown by the maximal real part of the eigenvalue of the linearized RNN state update around each interpolated fixed point location ('Analysis of fixed points for interpolated inputs' in Methods) (Fig. 4f). We interpret this result to mean that both category tasks reuse the unstable fixed point to move the state away from the category boundary.

The DelayAnti and ReactPro tasks were an example pair that did not share any dynamical motifs (Fig. 4g–i). The DelayAnti task began with the context period, followed by a stimulus presentation that signaled the opposite response direction (*φ*_response_ *=* *θ*_stimulus_ + π), followed by a ‘go’ cue that signaled when to initiate a delayed response (see Extended Data Fig. 1 for all task definitions). The ReactPro task began with the context period, followed by a stimulus presentation that signaled the same response direction and required an immediate response. During the context period, the network state evolved toward dissimilar locations for trials of either task, and trajectories during the stimulus period were not aligned (Fig. 4c, ‘Different Motifs’). We defined the subspace for the ReactPro task by performing PCA on the state trajectories during the stimulus period. We projected the DelayAnti task in the same subspace and found that little variance was captured by the other task PCs (Fig. 4g). We also visualized both tasks in a subspace defined by the first two PCs of the DelayAnti task and again found little overlap, suggesting that both tasks evolved in mostly non-overlapping subspaces (Fig. 4h). We interpolated across these two rule inputs during the stimulus period, revealing that there was a bifurcation where the relevant stimulus-dependent fixed point did not form a continuous bridge across interpolated rule inputs (Fig. 4h, middle). We quantified the distance between consecutive fixed points that were closest to the endpoint of the state trajectory for each interpolated input and identified a large discrete jump in the location of the relevant fixed point (Fig. 4i, right). We visualized the maximal real part of the eigenvalues of the linearized RNN state update around each consecutive fixed point, revealing qualitatively dissimilar local dynamics around fixed points for interpolated input conditions, indicated by crossing the stability threshold at one for this discrete dynamical system (Fig. 4i, left).

Taken together, these features suggest that shared dynamical motifs are implemented by evolving the state to the appropriate region of state space such that it interacts with a shared fixed point across similar task computations. Category tasks shared both unstable and stable fixed points. On the other hand, stimulus period dynamics for the DelayAnti and ReactPro tasks evolved in separate subspaces and were governed by different stable fixed points. These analyses revealed that shared structure was not merely an artifact of all tasks within the same network; rather, only tasks with similar computations implemented shared dynamical motifs. Note, however, that we cannot rule out the possibility that an alternate path of interpolation between inputs might reveal shared fixed points. See 'Analysis of fixed points for interpolated inputs' in Methods and 'Discussion' for limitations of the interpolated inputs analysis.

### Dynamical motifs result in modular lesion effects

Yang et al.^21^ found that network lesions affected sets of tasks that shared computational features. For example, if the output of a particular cluster of units was set to zero, then all tasks involving a particular computation decreased their performance, whereas other tasks were unaffected. Their work left open the major question of why lesion effects were modular. We identified the cause of these modular lesion effects to be related to the underlying modular dynamical motifs that perform computation.

We examined the impact of lesioning clusters of units described in the variance matrix in Fig. 3a. Many unit clusters had high variance for a set of task periods with similar computations. For example, unit clusters a and c had high variance for memory and response task period clusters 10 and 9, respectively (Fig. 3a). Other unit clusters had high variance for Modality 2 stimulus periods (unit cluster k) or anti stimulus periods (unit cluster f), etc. We lesioned a cluster of units by setting the output of all units within the cluster to be zero throughout a given trial.

**Fig. 5:**
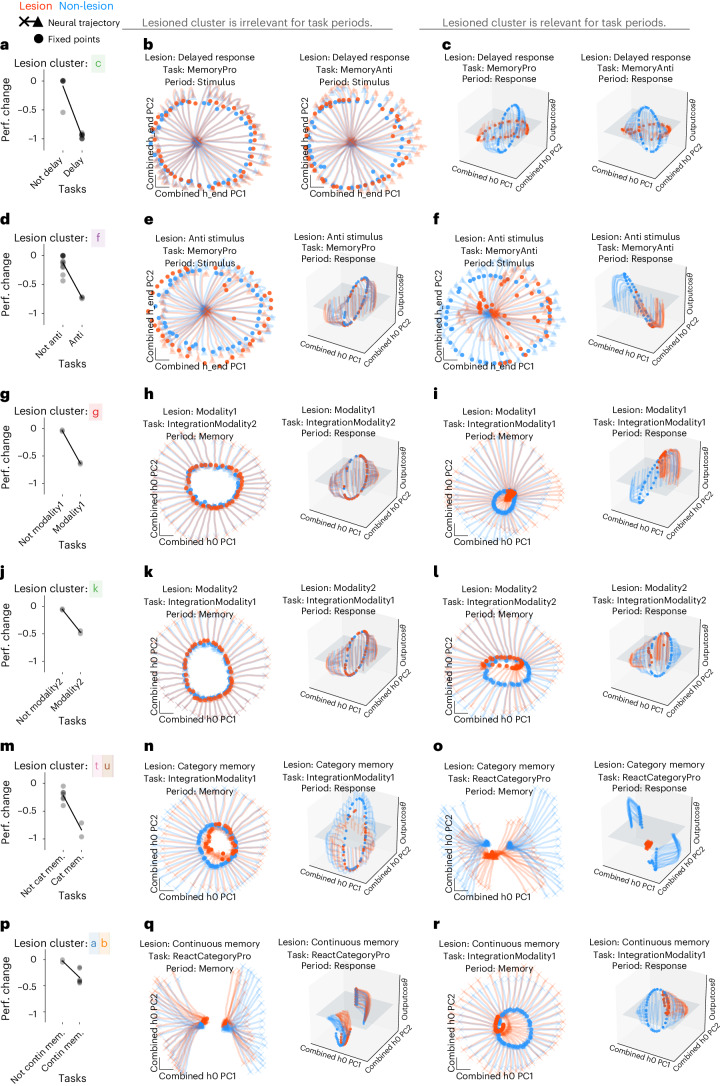
Unit cluster lesions had modular effects on task period clusters that shared the same dynamical motif. **a**–**c**, Delayed response lesion. **a**, Fraction performance change (each point is one task) after delayed response (cluster c in Fig. 3a) lesion by setting unit output to zero for units within cluster with high variance during the response period of delayed response tasks (see Supplementary Table 1 for motif definitions). **b**, Fixed points (filled circles) and state trajectories (starting from ‘x’ and ending with ‘▴’) during the performance of MemoryPro and MemoryAnti stimulus periods in lesioned (orange) and full (blue) network projected into the first two PCs (*x* and *y* axes) defined by the full and lesioned network state at the last timestep of the stimulus period. Stimulus period dynamics should not be relevant for the delayed response unit cluster lesion. As a result, fixed points and state trajectories diverge minimally from the full network. **c**, Same as **b** during the response periods in lesioned (orange) and full (blue) network projected into the first two PCs (*x* and *y* axes) defined by the full and lesioned network state at the first timestep of the response period and the output weight vector (from *W*_out_) associated with cos*θ*_stimulus_ on the *z* axis. Response period dynamics should be relevant for the delayed response unit cluster lesion. As a result, lesion prevents fixed points from rotating into output potent space and has a marked impact on network performance. **d**–**f**, Anti stimulus lesion. **d**, Same as **a** for anti stimulus (cluster f in Fig. 3a) during irrelevant MemoryPro stimulus and response task periods (**e**) and relevant MemoryAnti stimulus and response task periods (**f**). Fixed points and activity are projected onto the first two PCs defined by the network state at the last timestep of the stimulus period for both the lesioned and full network (*x* and *y* axes) (left) and the first timestep of the response period and the output weight vector (from *W*_out_) associated with cos*θ*_stimulus_ on the *z* axis (right). **g**–**i**, Modality 1 Lesion. **g**, Same as **a** for Modality 1 (cluster g in Fig. 3a). **h**, Irrelevant IntegrationModality2 stimulus and response task periods. **i**, Relevant IntegrationModality1 stimulus and response task periods. Fixed points and activity are projected onto the first two PCs (*x* and *y* axes) defined by the network state at the first timestep of the memory task period for both the lesioned and full network and the output weight vector (from *W*_out_) associated with cos*θ*_stimulus_ on the *z* axis. **j**–**l**, Modality 2 Lesion. **j**, Same as **a** for Modality 2 (cluster k in Fig. 3a). **k**, Irrelevant IntegrationModality1 stimulus and response task periods. **l**, Relevant IntegrationModality2 stimulus and response task periods. Fixed points and activity are projected onto the first two PCs (*x* and *y* axes) defined by the network state at the first timestep of the memory task period for both the lesioned and full network (left) and the first timestep of the response task period for both the lesioned and full network and the output weight vector (from *W*_out_) associated with cos*θ*_stimulus_ on the *z* axis (right). **m**–**o**, Category memory lesion. **m**, Same as **a** for category memory (clusters t and u in Fig. 3a). **n**, Irrelevant IntegrationModality1 memory and response task periods. **o**, Relevant ReactCategoryPro memory and response task periods. Fixed points and activity are projected onto the first two PCs (*x* and *y* axes) defined by the network state at the first timestep of the memory task period for both the lesioned and full network (left) and the first timestep of the response task period for both the lesioned and full network and the output weight vector (from *W*_out_) associated with cos*θ*_stimulus_ on the *z* axis (right). **p**–**r**, Continuous memory lesion. **p**, Same as **a** for continuous memory (clusters a and b in Fig. 3a). **q**, Irrelevant ReactCategoryPro memory and response task periods. **r**, Relevant IntegrationModality1 memory and response task periods. Fixed points and activity are projected onto the first two PCs (*x* and *y* axes) defined by the network state at the first timestep of the memory task period for both the lesioned and full network and the output weight vector (from *W*_out_) associated with cos*θ*_stimulus_ on the *z* axis. All panels were generated from the same network as in Fig. 3a with hyperparameters: LeakyRNN, 128 units, softplus activation, diagonal initialization. Perf., performance. h0 is the state at the beginning of the task period and h_end is the state at the end of the task period.

In six example lesions, we demonstrate that some unit cluster lesions impacted only a subset of tasks that shared computational features where units had high variance. Here, we show that lesions either did or did not impact task-relevant computations depending on whether the relevant underlying dynamical motif was impacted (Fig. 5). A lesion to one cluster (Fig. 3a, unit cluster c) only impacted performance on tasks that included a delayed response (Fig. 5a–c). This motif is responsible for rotating the ring attractor from output null space into output potent space^29^. We visualized fixed points and state trajectories for task periods where the delayed response motif was either irrelevant (stimulus period) or relevant (response period) during MemoryPro and MemoryAnti tasks in lesioned (orange) and non-lesioned (blue) networks (Fig. 5b,c). Fixed points and, in turn, state trajectories were markedly impacted when the delayed response dynamical motif was lesioned during the response period of the MemoryPro and MemoryAnti tasks but not during the stimulus period of either task. In a second example, we lesioned a cluster of units with high variance during the performance of anti response tasks (Fig. 3a, unit cluster f) and found that there was minimal change in the fixed points and state trajectories of pro response tasks (Fig. 5d–f). We present additional examples for Modality 1, Modality 2, Category Memory and Continuous Memory motifs (Fig. 5g–r). Note that our clustering approach produces an arbitrary threshold to separate branches of the dendrogram into different groups (Fig. 3a and 'Clusters' in Methods). As a result, we sometimes combined different clusters that were on the same branch in our lesion studies. For category memory and continuous memory tasks, we lesioned two adjacent unit clusters (t and u for category memory and a and b for continuous memory). This highlights limitations in our approach to link clusters of units directly to dynamical motifs, which will be an important direction for future studies.

When tasks did not use the dynamical motif impacted by a unit cluster lesion, they were not impacted by the lesion. For example, MemoryPro stimulus period activity was not impacted by a lesion to the unit cluster which implemented the response period dynamical motif of the same task (Fig. 5b). The state evolved toward a stable fixed point at approximately the same location in both the lesioned (orange) and full (blue) network after this cluster was lesioned. However, this lesion resulted in minimal rotation into output potent space during the response period (Fig. 5c, middle and right). Conversely, there was little change in the fixed point structure after a lesion to the anti stimulus motif (cluster f) for all task periods during the MemoryPro task (Fig. 5e). Taken together, results from our lesion studies suggest that modular lesion effects are a result of modular fixed point structures that implement dynamical motifs.

### Fast learning of novel tasks by reusing dynamical motifs

**Fig. 6:**
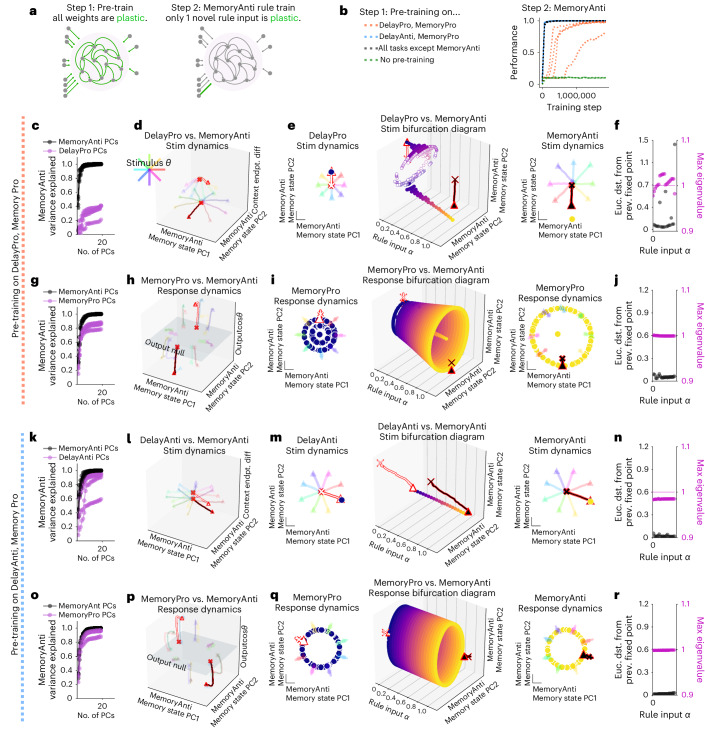
Dynamical motifs were reused for fast learning of novel tasks with familiar computational elements. **a**, Schematic of two-stage learning. Networks were pre-trained on a set of tasks while all weights were plastic. The same network was then trained on a novel task by only learning weights for a single new one-hot rule input. **b**, Left; networks were pre-trained on two tasks that include pro stimulus and continuous memory motifs (orange), anti stimulus and continuous memory motifs (blue), all motifs (black) and no motifs (green). See Supplementary Table 1 for motif definitions. Right: performance during MemoryAnti task rule input weight training after pre-training on various sets of motifs for five different networks each. **c**–**j**, Network pre-trained on DelayPro and MemoryPro tasks (pro stimulus and continuous memory motifs) was then trained to perform MemoryAnti through weight changes to the MemoryAnti rule input (*N*_rec_ × 1 vector). **c**, Fraction of MemoryAnti stimulus period variance explained by MemoryAnti stimulus period PCs (black) and DelayPro stimulus period PCs (purple) quantifies the extent to which both pro and anti tasks are in a similar subspace during the stimulus period. **d**, Stimulus period activity for DelayPro (white) and MemoryAnti (black) tasks for eight different stimulus angles (rainbow colors) projected into PC space (*x* and *y* axes) defined by state trajectories during MemoryAnti task and context period state endpoint difference between both tasks (*z* axis). **e**, Rule input interpolation across tasks for one stimulus angle. Middle: unstable (open) and stable (closed) fixed points for 20 intermediate *α* values (*x* axis) projected onto top two PCs defined by state trajectories during the memory period of the MemoryAnti task (*y* and *z* axes) with DelayPro *α* = 0 (left) and MemoryAnti *α* = 1 (right) fixed points and activity for eight different stimulus angles (rainbow colors). **f**, Euclidean distance between fixed points (black) and maximum real eigenvalue for the linearization of the state update around each fixed point (purple) for the single fixed point closest to the state at the end of the stimulus period for 20 consecutive *α* values between 0 and 1. Analyzing only one fixed point that is most proximal to the end of the state trajectory for each input condition ('Analysis of fixed points for interpolated inputs' in Methods). **g**–**j**, Same as **c**–**f** for response period of MemoryPro and MemoryAnti tasks. **k**–**r**, Network pre-trained on DelayAnti and MemoryPro tasks (anti stimulus and continuous memory motifs) was then trained to perform MemoryAnti task through weight changes to the MemoryAnti rule input (length *N*_rec_ vector). **k**–**n**, Same as **c**–**f** for stimulus period of DelayAnti and MemoryAnti tasks with pre-training on DelayAnti and MemoryPro tasks. **o**–**r**, Same as **c**–**f** for response period of MemoryPro and MemoryAnti tasks with pre-training on DelayAnti and MemoryPro tasks. **d**–**f** and **h**–**j** were generated from one network, and **l**–**n** and **p**–**r** were generated from a different network, both with hyperparameters: LeakyRNN, 256 units, softplus activation, diagonal initialization. Euc. dst., Euclidian distance.

**Extended Data Fig. 10:**
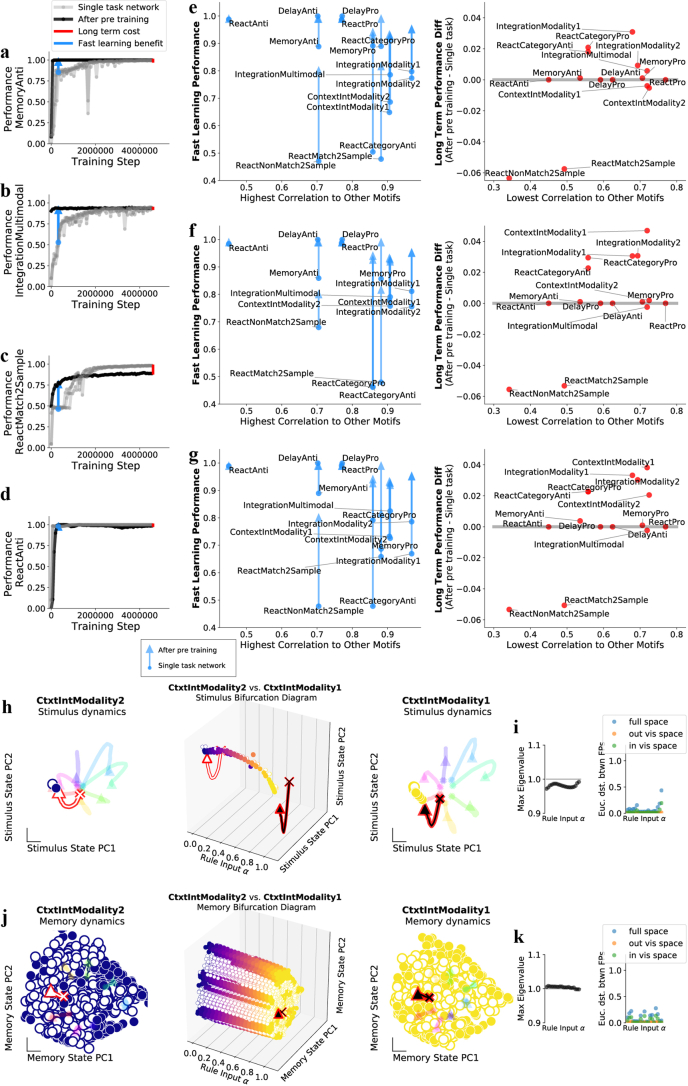
Leave-one-out transfer learning is possible through reuse of previously learned motifs and is therefore less effective for tasks with unique dynamical motifs. **(a-d)** Performance during training single rule input weights for **(a)** MemoryAnti **(b)** IntegrationMultimodal **(c)** ReactMatch2Sample and **(d)** ReactAnti tasks after pre-training all other weights and biases on all other tasks (black) and during training all weights and biases on a single rule (gray, 3 lines for 3 different networks with different random reeds). Single task networks provide a baseline comparison to measure how well the transfer learning method works for any given task. Notice transfer learning method typically results in faster learning (black line goes up before grey lines), except for ReactAnti task, which is rapidly learned in both cases. During training of the ReactMatch2Sample tasks, single task networks with fully plastic weights eventually out-perform transfer learning networks with only one plastic input. Through examination of the similarity of unit activations across tasks, we understand single task networks’ superiority over the transfer learning method to be a result of unique dynamical motifs in the held-out task (ReactMatch2Sample). **(e**) **left:** Performance early in training novel task for single task networks (dot) and transfer learning network (arrowhead) (as in **a-d**) plotted against task period max correlation (for a given task, plotting task period with the highest max correlation with other rows in the variance matrix, Fig. 3a). Task period max correlation was averaged across networks with all hyperparameter settings in Fig. 3b (see Transfer learning Methods for details). High correlation values on the x-axis highlight that all tasks (except ReactAnti) have at least one task period with high correlation (>.5) to a task period from a different task, and therefore benefit from pre-training to some extent (arrowhead above dot in all cases). Some tasks are so easy (DelayPro, DelayAnti, ReactPro and ReactAnti) that performance is nearly 100% in both transfer learning and single task networks at the early training step, resulting in zero performance difference. **right**: Performance difference (transfer learning performance - single task network performance) late in learning plotted against task period max correlation for the task period with the lowest value in each task. The x-axis shows which tasks include task periods with novel dynamical motifs indicated by low correlation to task periods in other tasks (for a given task, plotting task period with the lowest max correlation with other rows in the variance matrix, Fig. 3a). **(f-g)** Same as **e** for other single task networks with different random seeds. ReactMatch2Sample and ReactNonMatch2Sample tasks both have a task period with low correlation to other task periods from different tasks and therefore ReactMatch2Sample and ReactNonMatch2Sample tasks require unique dynamical motifs. Those were the same tasks where single task learning outperformed the transfer learning approach. This suggests our transfer learning approach depends on pre-training necessary motifs. **(h-k)** Additional support that leave-one-out transfer learning is possible through reuse of previously learned motifs: These figures present additional support for examples in the main text (Fig. 6). We first train on all tasks except ContextIntegrationModality2 and then use our transfer learning approach to learn the context inputs for the held-out task. Same stimulus fixed point was reused across ContextIntegrationModality1 and 2 tasks. Memory period reused the same plane attractor to store the amplitude information of the stimulus. See Extended Data Fig. 9 for detailed fixed-point structures on the integration tasks. Input interpolation between fixed points across ContextIntegrationModality1 and ContextIntegrationModality2 tasks during (**h-i**) ‘Stimulus Integration’ and (**j-k**) ‘Integration Memory’ motifs for **(h)** Rule input interpolation across context integration tasks for one stimulus angle during first stimulus period. **middle**: Unstable (open) and stable (closed) fixed points for 20 intermediate *α* values (x-axis) projected onto top two PCs defined by state trajectories during the stimulus period both tasks concatenated (y and z-axes) with (**left**) ContextIntegrationModality2 *α*=0 and (**right**) ContextIntegrationModality1 *α*=1 fixed points and trajectories for 8 different stimulus angles (rainbow colors). **(i) left**: Maximum real eigenvalue for the linearization of the state update around each fixed point single fixed point closest to the state at the end of the stimulus period for 20 consecutive *α* values between 0 and 1. **right**: Euclidean distance between consecutive fixed points in the full state space (blue), outside of the visualized subspace in **h** (orange) and within the visualized space (green). We analyzed only one fixed point that is most proximal to the end of the state trajectory for each input condition to highlight the task relevant fixed point (see Analysis of fixed points for interpolated inputs in Methods). **(j-k)** Same as **h-i**, but for memory period. Panels **h-k** generated from the same network. All networks in **a-g** were trained with the same hyperparameters: LeakyRNN, *softplus* activation, diagonal initialization, *N*_rec_ = 128 for transfer learning and single task networks.

Networks were able to rapidly learn new tasks sequentially by reconfiguring previously learned dynamical motifs. We first identified a task where each task period shared a dynamical motif with at least one of the other 14 tasks: MemoryAnti. This task requires the anti stimulus motif (Fig. 2d) and the delayed response memory motif (Figs. 1e–h and 2f–i). We next trained a network to perform every task except the MemoryAnti task. After learning all other tasks, we trained only the *N*_rec_-dimensional rule input vector for the MemoryAnti task (Fig. 6a). By training only one new set of rule input weights, we did not interfere with any previously learned dynamical motifs within the recurrent weight matrix, *W*_rec_, enabling learning of new tasks without catastrophic forgetting. The network was able to learn the MemoryAnti task when previously trained on all other tasks (Fig. 6b, black). Similar results were observed for other tasks (Extended Data Fig. 10a–d).

To determine if the anti stimulus and delayed response dynamical motifs were sufficient, we pre-trained a network on tasks containing these motifs (DelayAnti and MemoryPro). Pre-training on this minimal set with relevant motifs achieved similar speed and proficiency as pre-training on all tasks (Fig. 6b, blue), suggesting that these motifs were sufficient. Conversely, pre-training on tasks lacking the anti motif (orange) or no pre-training (green) resulted in significantly slower learning or failure to learn MemoryAnti entirely (Fig. 6b).

Despite slow and variable learning, networks pre-trained on tasks lacking the anti motif were still able to learn the new dynamical motif by modifying the rule input vector (Fig. 6b, orange). This resulted in stimulus period state trajectories that were not highly overlapping with previously learned tasks (Fig. 6c). Linear interpolation between DelayPro and MemoryAnti rule inputs revealed a qualitative change in the dynamics across input conditions. This suggests that the MemoryAnti stimulus period relevant fixed point was distinct from the previously learned DelayPro stimulus period relevant fixed point (Fig. 6e,f). Despite learning a new stimulus period anti motif, the network was still able to reuse the previously learned memory motif (Fig. 6g–j). This result highlights the modularity of dynamical motifs.

Networks that were pre-trained with the relevant dynamical motifs reused the anti stimulus and memory dynamical motifs for fast learning of the novel MemoryAnti task. MemoryAnti state trajectories were in highly overlapping subspaces with the DelayAnti state trajectories during the stimulus period (Fig. 6k,l) and with the MemoryPro state trajectories during the memory period (Fig. 6o,p). Rule input interpolation between both anti tasks during the stimulus period (Fig. 6m) and memory tasks during the response period (Fig. 6q) provided strong evidence that the fixed point structures were shared.

In another example, we first trained on all tasks except ContextIntegrationModality2 and then used our transfer learning approach to learn the context inputs for the held-out task (Extended Data Fig. 10h–k). We found that the same stimulus fixed point was reused across ContextIntegrationModality1 and ContextIntegrationModality2 tasks (Extended Data Fig. 10h,i). This fixed point shifted in state space corresponding to the relevant modality. The same plane attractor was reused to store the amplitude information of the stimulus throughout the memory period (Extended Data Fig. 10j,k). The requirement for these tasks to compare the amplitude of two consecutive stimuli resulted in a plane attractor rather than the ring attractor in other tasks, which has no amplitude information. See Extended Data Fig. 9 for detailed fixed point structures that implement integration tasks.

We wanted to better understand how transfer learning effectiveness relates to the uniqueness of a task’s dynamical motifs. We trained networks on all but one task each, followed by training only the rule input for the held-out task. Performance was compared to single-task training (Extended Data Fig. 10). In Fig. 3, we calculated the correlation between rows in the variance matrix to identify task periods that might share dynamical motifs. In the following analysis, we used correlation between rows in the variance matrix to identify which tasks might benefit from pre-training on other tasks (see 'Transfer learning' in Methods for details).

Nearly all tasks shared similarities with others (high correlation) and benefited from pre-training early in training (faster performance gain) (Extended Data Fig. 10a–d and 10e–g, left). Tasks with unique dynamical motifs had at least one task period where the maximum correlation to other task periods was low (ReactAnti, ReactNonMatch2Sample and ReactMatch2Sample). These tasks could not be learned as well using this pre-training method compared to full network training, except for ReactAnti, which was easily learned by either training approach (Extended Data Fig. 10d and 10e–g). In summary, we found that rapid learning was not as successful in the context of novel tasks with unique dynamical motifs. These results provide support that rapid learning of novel tasks requires reconfiguration of relevant previously learned dynamical motifs.

## Discussion

In this work, we examined how recurrently connected artificial networks flexibly repurposed their learned dynamics to perform multiple tasks. Our collection of commonly studied cognitive tasks could be broken down into an underlying set of subtasks (contextual integration, memory, categorization, anti-response, etc.; see Supplementary Table 1 for all motif definitions). We showed that networks learned this underlying subtask structure, which resulted in specialized computational building blocks that we call dynamical motifs, dedicated to each subtask. Using input interpolation and fixed point analyses, we showed how dynamical motifs were organized in relation to one another and often shared across tasks or task periods. Inputs reconfigured the dynamical system in each task period, often resulting in smooth changes to the dynamical landscape underlying the performed computation. Motifs necessary to perform each subtask included different types of attractor structures, input amplifications, decision boundaries and rotations. The modular subtask structure in our set of tasks is analogous to the structure of language, mathematics and other natural behaviors in everyday life^31,32^.

Our framework of examining subtask computation through the lens of dynamical motifs made it possible to explain lesions and learning results described previously^19,21^. As in the study by Yang et al.^21^, we found that lesioning specific unit clusters resulted in specific deficits in sets of tasks that were related computationally. Units within a cluster had high variance during a set of task periods that shared a dynamical motif. When we lesioned a given unit cluster, the fixed points that made up the associated dynamical motif were greatly impacted in terms of their locations and stability. A unit cluster associated with one dynamical motif could be lesioned with little impact to other computations that the network performed. This finding was surprising given the all-to-all connectivity possible in our networks as well as the fact that no regularizations or constraints to induce modularity were employed in the training of the RNNs. Recent work on subpopulation structure for the implementation of multiple tasks provides insight for these findings^22,33^.

We demonstrated that networks equipped with relevant dynamical motifs could repurpose those motifs modularly for fast learning of novel tasks. The initial phase of learning novel dynamical motifs was a slow process. However, given a rich repertoire of previously learned motifs, a network could quickly repurpose motifs to perform novel tasks by learning a single input weight vector. Our findings suggest that a useful lifelong learning strategy could include two stages of learning. Early in learning, it may be beneficial for highly plastic recurrent connections throughout the brain to learn novel subtasks (dynamical motifs). Late in learning, reduced plasticity in recurrent connections and new plastic layers that function as contextualizing inputs could repurpose previously learned subtasks. This hypothesis is interesting to consider in the context of critical periods^34^ and re-aiming^35^. We hypothesize that this two-stage process of slow and fast learning could provide some intuition for off-manifold and on-manifold brain–machine interface learning results in non-human primates^36–38^. Reusable dynamical motifs may inform state-of-the-art models that require pre-training^39^.

Our results are based on artificial systems, lacking the complexities of real brains. We used simplified networks without diverse cell types or prescribed architectures and only applied noisy static inputs. Although our learning rules are not biological, we hypothesize that optimized artificial neural networks and the principles that we uncover from them are informative about biological neural circuits based on principles of optimality and robustness^40^. Although some constraints changed the way dynamical motifs were shared, our main finding that they are, in fact, shared was robust across all types of networks and hyperparameter choices that we tested, including large networks without noise (Extended Data Fig. 3e–l). These findings suggest that shared motifs are not a result of limited computational resources. We hypothesize that the modular organization of dynamical motifs was a result of the modular subtask structure of our tasks, but learning dynamics through gradient descent could play a role^41^. It will be of great interest to further explore the prevalence of dynamical motifs in other artificial^42^ and biological systems^43^.

We examined a set of cognitive tasks commonly studied in humans and other animals^21,22,25^. Networks learned a variety of fixed point structures capable of implementing these computations. Notably missing from this set of attractors were limit cycles, which have been shown to be important for computations that require specific timed responses. Additionally, tasks involving complex pattern generation or continuously changing inputs, such as real-time adaptation or tracking, may involve dynamics that are not well captured by our fixed point analysis. These limitations highlight the versatility of RNNs and the need for further exploration with a broader range of tasks. Fixed point structures often moved in different contexts rather than appeared or disappeared (Figs. 1g, 2f,h and 3d,e). This is consistent with previous work, where graded input results in a continuum of stable fixed points^15,44^. Interpolations across contextual inputs, with intermediate values that the network was never exposed to during training, revealed an absence of bifurcations in the task-relevant dynamical motif. This finding suggested that motifs were reused across tasks and presents a conceptual advance for thinking about the relationship between different computations. Future research should explore the connection among high-dimensional parameter bifurcations, composition and computation^45–48^.

Fixed points often persisted even when the inputs were not relevant to the current task (Fig. 2c). These irrelevant fixed points did not interfere with network computation because the network state was organized to be more proximal to task-relevant fixed points. This aligns with the concept of ‘sloppiness’ in flexible systems^49–51^.

In this work, we focused on dynamical motifs that share fixed points across tasks because we could measure this feature through empirical bifurcation diagrams. However, tasks that do not share exactly the same fixed points could still evolve on a similar, nearby dynamical landscape. Notice that, in Fig. 4c, there are not two discrete clusters; rather, we found a continuous distribution of angle between trajectories and proximity of initial conditions. Recently developed analysis tools that quantify similarity of dynamical systems could lay the groundwork for studying shared dynamical motifs without fixed points^52,53^.

Our linear interpolation analysis presents limitations in identifying shared motifs. We assert that motifs are shared if interpolation reveals a smooth fixed point transition with no detected bifurcations. However, if we identify a bifurcation along one path, alternative interpolation paths might reveal continuous transitions. Examining all paths is impractical, so we cannot definitively claim that two motifs are not shared through this approach.

Compositional systems require modularity to recombine components in different contexts, fostering flexibility and generalization. We identified dynamical motifs as the underlying modular substrate that could support compositional computation. Previous work demonstrated that generalization to new tasks could be implemented without training by using a linear combination of task inputs^21^. We demonstrate that learning a single input is sufficient to link previously learned motifs within a fixed recurrent network. It will be of great interest to identify how and why a linear combination of inputs is able to recruit combinations of previously learned motifs. This will require a better understanding of how motifs are sequentially recruited and how inputs might reconfigure the space between and around fixed points that implement computation. We found that similar computations are proximal to each other in state space (Fig. 4b). This results in a hierarchical organization of tasks based on similarity (as shown in the variance matrix) and begins to address the question of compositionality. Although we think that this smoothness in state space^14,17,54^ is related to compositionality, it remains for future work to tackle head on why linear combinations of previously unseen task inputs should result in largely functioning systems.

Our findings offer several testable predictions. The method of studying unit variance across tasks could be readily performed on neural data (Fig. 3a). This analysis could be informative about perturbations to biological network activity that would most markedly impact performance on a computation of interest (Fig. 5). Results might apply across biological scales, from multiple motifs within a brain region to distinct computations in different cortical areas. Our approach for training networks sequentially by reusing previously learned dynamical motifs could be used to determine ideal curricula for training animals on complex tasks. For example, given a particular task of interest, one could train an artificial network to perform the task and inspect all relevant dynamical motifs. For example, the ‘anti’ and ‘memory’ motifs were the sufficient set of relevant motifs for the MemoryAnti task in Fig. 6b. Based on the task-relevant motifs, one could systematically design a set of tasks to learn a sufficient set of motifs rather than designing a curriculum through guesswork. Additionally, this work highlights the relevance of reporting training protocols as they may shape the dynamical motifs that implement computation^55–57^. Beyond experimental predictions, our work provides intuition for why the brain exhibits functional specialization.

Through the lens of dynamical systems, we identified the underlying computational substrate for clustered representations described previously by Yang et al.^21^ and highlighted a new level of organization between the unit and the network: groups of units that implement dynamical motifs. More broadly, our findings highlight the relevance of dynamical systems as a framework to better understand the response properties of neurons in the brain. As researchers record more whole-brain activity, the framework of dynamical motifs will guide questions about specialization and generalization across brain regions.

## Methods

### Network structure

We examined ‘vanilla’ continuous-time RNNs for most of this work, although see 'Alternative hyperparameters and network architectures' in Methods on varying hyperparameters and architectures. Before time discretization, RNN network activity ${\bf{h}}$, a vector of length *N*_rec_, followed the dynamical equation

4 $$\tau \frac{{\rm{d}}{\bf{{h}}}}{{\rm{d}}t}(t)=-{\bf{h}}(t)+\sigma ({\bf{i}}(t))$$

with the total neuron input ${\bf{i}}(t)$ defined as

5 $${\bf{i}}(t)\equiv {W}_{{\rm{rec}}}\;{\bf{h}}(t)+{W}_{{\rm{in}}}\;{\bf{u}}(t)+{\bf{b}}_{{\rm{in}}}+{\bf{\upxi}} (t).$$

*W*_in_ and *W*_rec_ were the input and recurrent connection matrices of size *N*_rec_ × *N*_in_ and *N*_rec_ × *N*_rec_. These matrices specified the contribution of the inputs and upstream network activity to downstream network activity. The bias vector, ${\bf{b}}_{{\rm{in}}}$, was of length *N*_rec_. The private noise, **ξ**(*t*), was *N*_rec_ independent Gaussian white noise processes with zero mean and standard deviation of 0.158. The state of this system evolved over time according to the current state ${\bf{h}}(t)$ and inputs to the system ${\bf{u}}(t)$. The nonlinear function *σ*(·) was chosen to be softplus, tanh or retanh (see 'Alternative hyperparameters and network architectures' for details). The time constant, *τ*, specified the rate of decay of the network state. After using the first-order Euler approximation with a time-discretization step Δ*t*, we had

6 $${\bf{h}}_{t+1}=(1-\gamma )\;{\bf{h}}_{t}+\gamma \sigma ({\bf{i}}_{t}),$$

where *γ* ≡ Δ*t*/*τ*, which we set to 0.2. The full update equation was

7 $${\bf{h}}_{t+1}=(1-\gamma )\;{\bf{h}}_{t}+\gamma \sigma ({W}_{{\rm{rec}}}\;{\bf{h}}_{t}+{W}_{{\rm{in}}}\;{\bf{u}}_{t}+{\bf{b}}_{{\rm{in}}}+{\bf{\upxi}} _{t}).$$

A set of output units **z** was read out from the network according to

8 $${{\bf{z}}}_{t}={W}_{{\rm{out}}}{{\bf{h}}}_{t}+{\bf{b}}_{{\rm{out}}}.$$

*W*_out_ was the output connection matrix of size *N*_out_ × *N*_rec_, and ${\bf{b}}_{{\rm{out}}}$ was a bias vector of length *N*_out_. All *W* matrices, *W*_in_, *W*_rec_, *W*_out_ and bias vectors ${\bf{b}}_{{\rm{in}}}$, ${\bf{b}}_{{\rm{out}}}$ were learned over the course of training (see 'Training procedure' for details).

The network received four types of noisy input.

9 $${\bf{u}}=[{{u}}_{{\rm{fix}}},{{\bf{u}}}_{{\rm{mod1}}},{{\bf{u}}}_{{\rm{mod2}}},{{\bf{u}}}_{{\rm{rule}}}]+{{\bf{u}}}_{{\rm{noise}}}$$

10 $${\bf{u}}_{{\rm{noise}}}[j] \sim \sqrt{0.1}\,\mathcal{N}(0,1)$$

The fixation input *u*_fix_ was 1 when the network was required to fixate and 0 when the network was required to respond. The stimulus inputs ${\bf{u}}_{{\rm{mod1}}}$ and ${\bf{u}}_{{\rm{mod2}}}$ each had a length-2 vector of (*A*sin*θ* and *A*cos*θ*) representing a different ‘modality’ and each modality representing a one-dimensional circular variable described by the degree around a circle. The strength of the stimulus inputs varied in amplitude according to *A*. We greatly reduced the dimensionality of the stimulus inputs from the original implementation of these tasks to simplify visualizations and analysis^21^. This simplification in stimulus inputs required removal of five of the original 20 tasks, because we could no longer present multiple stimuli in the same modality simultaneously. The network also received a set of rule inputs encoded in the vector ${\bf{u}}_{{\rm{rule}}}$. This vector represented which task the network was supposed to perform on each trial as a one-hot vector. The rule input unit corresponding to the current task was 1, whereas other rule input units were 0. Therefore, the number of rule input units was equal to the number of tasks trained. The rule unit activation patterns for different rules were orthogonal to each other in this one-hot encoding. Therefore, relationships between tasks were learned by the network rather than baked into the inputs. Finally, each input had Gaussian noise added to it according to equations (9) and (10). Here, the input noise strength was scaled by the factor $\sqrt{0.1}$. Note that this was an order of magnitude greater than in previous work, to prevent over-fitting^21^.

In total, there were

*N*_in_ = 1(fixation) + 2(modalities) × 2(*A*sin*θ* and *A*cos*θ*) + 15(rule) = 20 input units.

The network projected the state, ${\bf{h}}_{t}$, to an output ring, which contained two units (sin*ϕ* and cos*ϕ*) to encode response direction on a circle. In addition, the network projected ${\bf{h}}_{t}$ to a fixation output unit, which should be at the high activity value of 1 before the response and at 0 once a response is generated.

In total, there were

*N*_out_ = 1(fixation) + 1(modality) × 2(sin*ϕ* and cos*ϕ*) = 3 output units.

### Tasks and performances

Inputs and outputs for an example trial on each task are shown in Extended Data Fig. 1. Fixation input was 1 for the duration of the trial until the response period, when fixation input changed to 0. Reaction timed tasks never received a ‘go’ cue; therefore, the fixation input was always at 1, and the network was required to break fixation to respond as soon as the relevant stimulus arrived. Target fixation output activity was high (${\hat{z}}=0.8$) before the response period and low (${\hat{z}}=0$) during the response period for all tasks. If the activity of the fixation output prematurely fell below 0.5, the network was considered to have erroneously broken fixation, and the trial was incorrect.

The response direction of the network was read out in a two-dimensional vector (sin*ϕ* and cos*ϕ*). The decoded response direction was considered correct if it was within π/10 of the target direction.

Initial state for trials was randomly generated on each task from the same random seed, unique to each network. Therefore, across different tasks, random initial conditions for each trial were identical.

Tasks could be divided into periods, where each task period was a segment of sequential timesteps with continuous inputs (excluding noise). Each set of inputs reconfigured the network into a new dynamical landscape, with a different fixed point structure. Distinct dynamical landscapes for each input condition is a crucial concept in dynamical systems and should be emphasized^27^. For all tasks, in the first period (context), the rule input provided the network with information about task context. The onset of stimulus information marked a change in the stimulus inputs and the beginning of the next task period (stimulus1). All tasks had at least one stimulus period, but some had two stimulus periods. The period between the stimulus and response or between two stimuli was the memory period (memory1). If there was a second stimulus (stimulus2), sometimes the network was required to respond immediately to the second stimulus, and, in other tasks, there was an additional memory period (memory2) before the response period (response). The duration of the context, stimulus1, memory1, stimulus2, memory2 and response periods were *T*_context_, *T*_stimulus1_, *T*_memory1_, *T*_stimulus2_, *T*_memory2_ and *T*_response_, respectively. We adjusted the distribution of task periods to be wider than in previous work and drawn from a uniform distribution to prevent the network from predicting task period transitions. These modifications had a simplifying effect on fixed point structures.

*U*(*t*_1_, *t*_2_) is a uniform distribution between *t*_1_ and *t*_2_. The unit for time is milliseconds (timesteps × Δ*t*). Stimuli were presented in either modality 1 or modality 2 at random unless stated otherwise.

#### Delayed response

(Two tasks) DelayedPro: Move in same direction as stimulus (*ϕ*_response_ = *θ*_stimulus_) after delay. DelayedAnti: Move in opposite direction as stimulus (*ϕ*_response_ = *θ*_stimulus_ + π) after delay. Stimulus remains on throughout stimulus and response periods.

$$\begin{array}{l}{T}_{{\rm{context}}}\,U{(300,700)}\\ {T}_{{\rm{stim1}}}\,U{(200,{\mbox{1,500}})}\\ {T}_{{\rm{response}}}\,U{(300,700)}\end{array}$$

#### Memory response

(Two tasks) MemoryPro: Move in same direction as stimulus (*ϕ*_response_ = *θ*_stimulus_) after memory. MemoryAnti: Move in opposite direction as stimulus (*ϕ*_response_ = *θ*_stimulus_ + π) after memory. Stimulus disappears for memory period and remains off during response.

$$\begin{array}{l}{T}_{{\rm{context}}}\,U{(300,700)}\\ {T}_{{\rm{stim1}}}\,U{(200,{\mbox{1,600}})}\\ {T}_{{\rm{memory1}}}\,U{(200,1600)}\\ {T}_{{\rm{response}}}\,U{(300,700)}\end{array}$$

#### Reaction timed

(Two tasks) ReactPro: Move in same direction as stimulus (*ϕ*_response_ = *θ*_stimulus_) immediately. ReactAnti: Move in opposite direction as stimulus (*ϕ*_response_ = *θ*_stimulus_ + π) immediately.

$$\begin{array}{l}{T}_{{\rm{context}}}\,U{(500,{\mbox{2,500}})}\\ {T}_{{\rm{stim1/response}}}\,U{(300,{\mbox{1,700}})}\end{array}$$

#### Decision-making

(Five tasks) Move in direction of stimulus with largest amplitude. IntegrationModality1: Only Modality 1 is presented. IntegrationModality2: Only Modality 2 is presented. ContextIntModality1: Both modalities presented, only attend Modality 1. ContextIntModality2: Both modalities presented, only attend Modality 2. IntegrationMultimodal: Both modalities presented, attend both modalities equally.

$$\begin{array}{l}{T}_{{\rm{context}}}\,U{(200,600)}\\ {T}_{{\rm{stim1}}}\,U{(200,{\mbox{1,600}})}\\ {T}_{{\rm{memory1}}}\,U{(200,{\mbox{1,600}})}\\ {T}_{{\rm{stim2}}}\,U{(200,{\mbox{1,600}})}\\ {T}_{{\rm{memory2}}}\,U{(100,300)}\\ {T}_{{\rm{response}}}\,U{(300,700)}\end{array}$$

#### Delay match

(Four tasks) Immediately move in direction of *θ*_stim2_ if sequentially presented pair match. ReactMatch2Sample: Match if same angle (*θ*_stim1_ = *θ*_stim2_). ReactNonMatch2Sample: Match if opposite angle (*θ*_stim1_ = *θ*_stim2_ + π). ReactCategoryPro: Match if same category $({\theta }_{{\rm{stim1}}},{\theta }_{{\rm{stim2}}} < \uppi )$ or $({\theta }_{{\rm{stim1}}},{\theta }_{{\rm{stim2}}} > \uppi )$. ReactCategoryAnti: Match if opposite category $({\theta }_{{\rm{stim1}}} < \uppi \,{\rm{and}} \,{\theta }_{{\rm{stim2}}} > \uppi )$ or $({\theta }_{{\rm{stim1}}} > \uppi \,{\rm{and}} \,{\theta }_{{\rm{stim2}}} < \uppi )$.

$$\begin{array}{l}{T}_{{\rm{context}}}\,U{(200,600)}\\ {T}_{{\rm{stim1}}}\,U{(200,{\mbox{1,600}})}\\ {T}_{{\rm{memory1}}}\,U{(200,{\mbox{1,600}})}\\ {T}_{{\rm{stim2/response}}}\,U{(300,700)}\end{array}$$

### Training procedure

The loss *L* to be minimized was computed by time averaging the squared errors between the network output ${\bf{z}}(t)$ and the target output ${\hat{\bf{z}}}(t)$.

$$L={L}_{{\rm{mse}}}\equiv {\left\langle {m}_{i,t}({\bf{z}}_{i,t}-{\hat{\bf{z}}}_{i,t})^{2}\right\rangle }_{i,t}$$

Here, *i* was the index of the output units, and *t* was the index for time. We implemented a mask, *m*_*i*,*t*_, for modulating the loss with respect to certain time intervals. For example, in the first 100 ms of the context and response periods, there was a grace period with *m*_*i*,*t*_ = 0. During the response period, *m*_*i*,*t*_ = 5, and for the rest of the trial, *m*_*i*,*t*_ = 1. For the fixation output unit, *m*_*i*,*t*_ was two times stronger than the mask for the *ϕ*_response_ output units. The training was performed with Adam, a variant of stochastic gradient descent^58^. The learning rate ranged from 10^−4^ (tanh networks) to 10^−3^ (all other networks). The decay rate for the first and second moment estimates were 0.9 and 0.999, respectively. During training, we randomly interleaved all the tasks with equal probabilities, except for the ContextIntModality1 and ContextIntModality2 tasks that appeared five times more frequently to prevent the network from integrating both modalities equally. This alternative strategy gave the network an accuracy close to 75% if trials from these tasks were not overrepresented. During training, we used mini-batches of 64 trials. In a given minibatch, all trials were generated from the same task for computational efficiency. Training was terminated when *L* stopped decreasing, which was generally after 5 × 10^7^ training steps.

The network and training were implemented in TensorFlow.

### Alternative hyperparameters and network architectures

We trained networks with the following possible hyperparameters and architectures:

The network architecture was either the leaky RNN architecture defined previously or the leaky GRU architecture^59^.

We explored a number of nonlinear functions *σ*(·):

11 $$\begin{array}{l}\,{\rm{softplus}}{:}\,\sigma (x)=\ln(1+{{\mathrm{e}}}^{\,x})\\ \,{\rm{retanh}}{:}\,\sigma (x)={\rm{max}}({\rm{tanh}}(x),0)\\ \,{\mathrm{tanh}}{:}\,\sigma (x)={\rm{tanh}}(x)=\frac{{{\mathrm{e}}}^{\,x}-{{\mathrm{e}}}^{\,x}}{{{\mathrm{e}}}^{\,x}+{{\mathrm{e}}}^{\,x}}\end{array}$$

We initialized each weight matrix from a diagonal matrix

12 $${W}_{{\rm{rec0}}}=g\,{I}_{{N}_{{\rm{rec}}}}$$

or from a random Gaussian

13 $${[{W}_{{\rm{rec0}}}]}_{ij} \sim \frac{g}{\sqrt{{N}_{{\rm{rec}}}}}\mathcal{N}(0,1).$$

Here, *g* scaled the values in the initial weights. In networks with the tanh activation function and the leaky RNN architecture, *g* = 1, and in all other networks, *g* = 0.8. We found that networks with the tanh activation function required this higher *g* value to prevent quenching network activity during training.

To avoid overly complex solutions that did not generalize well, we penalized high activity and strong weights using an L2 regularization on ${\rm{h}}$ and on each weight matrix *W*_in_, *W*_rec_ and *W*_out_. The hyperparameter selection criterion was the highest level of regularization that resulted in greater than 80% performance on held-out test data. We found the highest level regularization that still resulted in greater than 80% performance on all tasks to be 10^−6^ for both weight and activity regularization.

### Fixed points

Our networks were high-dimensional nonlinear systems, rendering them difficult to understand intuitively. Examination of these networks was made easier through analysis of fixed points, which are locations in state space where the motion of the system is approximately zero. Through a Taylor expansion of our dynamical equation, we may see that our nonlinear system can be approximated as a linear one around fixed points, ${\bf{h}}^{\ast }$:

14 $$\begin{array}{l}\frac{d{\bf{h}}}{\rm{dt}}=F({\bf{h}},{\bf{u}})\\ F({\bf{h}}^{\ast }+\delta\; {\bf{h}},{\bf{u}}^{\ast }+\delta\; {\bf{u}})\approx F({\bf{h}}^{\ast},{\bf{u}}^{\ast })+\frac{\partial F}{\partial {\bf{h}}}({\bf{h}}^{\ast },{\bf{u}}^{\ast })\delta {\bf{h}}+\frac{\partial F}{\partial {\bf{u}}}({\bf{h}}^{\ast },{\bf{u}}^{\ast })\delta {\bf{u}}\end{array}$$

The second-order terms (not shown) are approximately zero because $\Vert \delta {\bf{h}}\Vert^{2}\approx 0$. The first term, $F({\bf{h}}^{\ast},{\bf{u}}^{\ast})$, is zero by definition of the fixed point, where ${{\bf{h}}}^{\ast}$ is the location where the update $F({\bf{h}}^{\ast},{\bf{u}}^{\ast})= 0$. For most of this work, with the exception of Extended Data Fig. 9, we hold input values to their constant value during a task period. We can, therefore, ignore the last term where $\delta {\bf{u}}=0$. Therefore, around fixed points, we can approximate our nonlinear dynamical systems as the linear system $\frac{d\bf{h}}{{\rm{d}}t}\approx \frac{\partial F}{\partial{\bf{h}}}({\bf{h}}^{\ast},{\bf{u}}^{\ast})\delta {\bf{h}}$.

Eigendecomposition of the matrix $\frac{\partial F}{\partial {\bf{h}}}({\bf{h}}^{\ast},{\bf{u}^{\ast}})$ reveals in which dimensions of state space the dynamics are contracting, expanding or are marginally stable (that is, neither contracting or expanding). Eigenvectors with an associated real part of the eigenvalue *λ* < 1 are contracting dimensions, *λ* > 1 are associated with expanding dimensions and *λ* ≈ 1 are marginally stable. At a fixed point that is contracting in every dimension, the state is at a basin of attraction. This is a particularly useful dynamical system for preparing an optimal initial condition for the next task period. Marginally stable dimensions are useful for integrating noisy pulses of stimulus information and for memory of a continuous variable. Saddle points are contracting in some dimensions and repulsive in other dimensions. Saddle points are useful for decision-making along the repulsive dimensions. Repulsive dimensions can additionally be useful for keeping the neural state away from a particular region of state space. For example, the neural state must remain outside of output potent space (orthogonal to the readout weights) until the response period.

To identify fixed points, we empirically optimized for a set of $\{\,{\bf{h}}^{{1\ast}},{\bf{h}}^{{2\ast}},\ldots \}$ satisfying $F(\,{\bf{h}}^{\ast},{\bf{u}}^{\ast})=0$ while defining ${\bf{u}}^{\ast}$ by holding the inputs ${\bf{u}}$ constant for each task period. Each different input condition reconfigured the RNN into a new dynamical landscape with a different set of fixed points. Therefore, each set of $\{\,{\bf{h}}^{\mathrm{1\ast}},{\bf{h}}^{\mathrm{2\ast}},\ldots \}$ was associated with a particular input ${\bf{u}}^{\ast}$. At the fixed point ${\bf{h}}^{\ast}$ with inputs ${\bf{u}}^{\ast}$, the update to the state at the next time point is zero, and, therefore, the state does not move away from this location. We used the term fixed point to include approximate fixed points, where the update is small on the timescale of our task. Our fixed points range between *q* = 10^−3^ and *q* = 10^−15^, where $q=\frac{1}{2}[{\bf{h}}^{\ast}-f({\bf{h}}^{\ast},{\bf{u}}^{\ast})][{\bf{h}}^{\ast}-f({\bf{h}}^{\ast},{\bf{u}}^{\ast})]$. We included a wide range of *q* values to best highlight relevant dynamics on a case-by-case basis.

We used the Fixed Point Finder package in TensorFlow^28^.

### Input interpolation

We examined how fixed point structures moved and changed stability as the dynamical system was reconfigured by different inputs. To do this, we interpolated across pairs of inputs and identified fixed points for each intermediate input condition. For input vectors ${\bf{u}}_{1}$ and ${\bf{u}}_{2}$, we identified fixed points for $\alpha {\bf{u}}_{1}+(1-\alpha ){\bf{u}}_{2}$, where *α* was varied between 0 and 1 in 0.05 increments.

### Analysis of fixed points for interpolated inputs

After input interpolation (see 'Input interpolation' for details), we wanted to compare fixed points across input conditions to track their positions and stability in high-dimensional space. However, there were often multiple fixed points, making it difficult to track an individual fixed point across input conditions. We focused on the fixed point closest to the state at the end of a task period of interest (except in Fig. 4e,f, where we focused on the closest unstable fixed point because it appeared more relevant to the nonlinear dynamics—the closest fixed point was stable and was also shared across tasks). Our reasoning was that if the state evolved toward a particular fixed point, it was likely relevant for computation. After identifying fixed points during rule input interpolation, we ran the network forward from the beginning of the context period for each interpolated rule input and identified the fixed point closest to the network state at the end of the task period of interest (stimulus or response period). We refer to this closest fixed point as the ‘relevant’ fixed point for a given interpolated input. We calculated the Euclidean distance between relevant fixed points associated with adjacent interpolated input conditions (*α*_1_, *α*_2_) as

15 $$\begin{array}{l}d({\alpha }_{1},{\alpha }_{2})={\left\Vert\, {\bf{h}}^{\ast}_{\,{\rm{relevant}}}({\alpha }_{1})-{\bf{h}}^{\ast}_{\,{\rm{relevant}}}({\alpha }_{2})\right\Vert }_{2}\end{array}$$

We also tracked the stability of the relevant fixed point for each interpolated input. To do this, we performed eigenvalue decomposition on the Jacobian of the RNN state transition function at the relevant fixed point

16 $$\begin{array}{l}\frac{\partial F}{\partial{\bf{h}}}(\bf{h},{\bf{u}})|_{{\bf{h}}={\bf{h}}^{\ast}_{{\rm{relevant}}}({\alpha }_{1}),{\bf{u}}=\,{\bf{u}}^{\ast }({\alpha}_{1})}\end{array}$$

The eigenvalue with the maximum real value is informative about whether the relevant fixed point is stable. By tracking the stability over input interpolation, we could identify bifurcations in the dynamical landscape.

To examine the relevant dynamical motif for a given task period, we defined a ‘relevant fixed point’ to be the fixed point closest to the state at the end of the task period. If the input interpolation between *α* = 0 and *α* = 1 resulted in approximately the same location of the relevant fixed point and approximately the same local dynamics around the relevant fixed point, then we defined the relevant fixed point as being functionally the same across inputs, and, therefore, the dynamical motif was shared across input conditions.

Alternatively, if the interpolation between *α* = 0 and *α* = 1 resulted in a bifurcation of the fixed point structure, then we defined the dynamical motifs to be distinct. We highlight that our definition of distinct motifs was limited in that a different path for consecutive input interpolation might not result in a bifurcation. It will be of great interest to explore ambiguous cases of shared and distinct motifs in future work.

### Effective input modulation

In previous work, it was identified that relaxation dynamics of the network state could contextually integrate stimulus inputs^11^. We identified some networks that additionally contextually amplified stimulus inputs (Extended Data Fig. 9). To deconstruct how the signal from one input is contextually amplified, we look at the first-order Taylor series approximation of the state update around a particular input, ${\bf{u}}^{\ast }$, and its associated fixed point, ${\bf{h}}^{\ast }$:

17 $$\begin{array}{l}\frac{\partial F}{\partial{\bf{h}}}({\bf{h}}^{\ast},{\bf{u}}^{\ast})\delta {\bf{h}}+\frac{\partial F}{\partial{\bf{u}}}({\bf{h}}^{\ast},{\bf{u}}^{\ast})\delta {\bf{u}}\end{array}$$

The network received contextual rule input during the context period and moved toward a stable fixed point. We took this stable fixed point during the context period to be the initial conditions for the subsequent stimulus period. We modeled the initial stimulus input in the $\Delta {{\bf{u}}}_{{\rm{stimulus}}}={{\bf{u}}}_{{\rm{stimulus}}}-{{\bf{u}}}_{{\rm{context}}}$ term without changing $\frac{\partial F}{\partial{\bf{u}}}({\bf{h}}_{{\rm{context}}},{\bf{u}}_{{\rm{context}}})$ at the context-dependent fixed point. We calculate the input response for each stimulus condition for trials spanning [0, 2π) by calculating the norm of the dot product:

18 $${\left\Vert \frac{\partial F}{\partial {\bf{u}}}\left({\bf{h}}_{{\rm{context}}},{\bf{u}}_{{\rm{context}}}\right){\Delta} {\bf{u}}_{{\rm{stimulus}}}\right\Vert }_{2}\mathrm{}$$

We found that there was task-specific amplification of stimulus modality inputs, where the Modality 1 (2) input response was larger for tasks where the network must respond according to Modality 1 (2).

### Task variance analysis

To examine the contributions of unit variance to computation in each task period, we used a modified version of the task variance analysis described previously^21^. We ran the network forward for a set of possible stimulus conditions on the task of interest. For example, in the delayed response tasks, we presented the network with trials where *θ*_stimulus_ ranged from [0, 2π). In the decision-making tasks, we ran the same network with *θ*_stimulus_ ranging from [0, 2π) and coherences ranging from 0.005 to 0.2. We then computed the variance across possible stimulus conditions for each unit on each task period through time. This was a deviation from previous work^21^ in two ways. First, we computed task period variance across stimulus conditions for all task periods separately because we considered each task period as a separate dynamical landscape. Second, we computed variance through time rather than averaging across time, because we were interested in the dynamics rather than static representations. Variance during the fixation period was low, so we excluded the fixation period from this analysis. Independent noise (**ξ** in equation (7)) was set to zero for this analysis to eliminate the effect of recurrent noise. This analysis was a useful method to uncover unit contributions to network computations because our networks were activity regularized. Given activity regularization, any deviations from zero activity were costly for the network to produce and, therefore, likely beneficial for task computation. The result of this analysis was a matrix composed of columns of units and rows of task periods, where each index quantified the participation of a given unit to the computation during a given task period. We refer to this matrix as the variance matrix.

Correlations between variance matrices were computed by first sorting task period rows according to one reference network. A correlation matrix for each network was computed by finding the Pearson correlation between rows of the variance matrix for that network separately. Each correlation matrix was compared to every other correlation matrix by first flattening the the upper triangle of entries in each correlation matrix. We then calculated the Pearson correlation between this vector and the same vector associated with each trained network. Both trained and untrained networks were compared to trained networks to determine whether the structure in trained networks emerged due to the input structure or due to learned dynamical motifs.

### Clusters

We sorted rows and columns of the variance matrix (see 'Task variance analysis' for details) according to similarity using the Ward variance minimization algorithm^60^. This algorithm produced a dendrogram that shows the hierarchical distance between rows or columns of the task variance matrix. We chose a hierarchical clustering algorithm rather than *k*-means, used in previous work^21^. We found that dynamical motifs appeared to be organized hierarchically, where the first division was between stimulus and response type computations. Further subdivisions separated more subtle differences, such as pro and anti stimulus motifs. The reader can see in the dendrogram how different thresholds might change the number of clusters. This finding aligns well with connectivity reported in cortical and thalamic brain regions^61^. We chose this visualization method rather than t-distributed stochastic neighbor embedding (t-SNE) because of the hierarchical structure of the data and because we find t-SNE difficult to interpret considering that embeddings are nonlinearly transformed.

To obtain discrete clusters, we identified the optimal distance threshold for each dendrogram by computing the silhouette score on the basis of intracluster and intercluster distances. The silhouette score of a unit *i* was $({d}_{i,{\rm{inter}}}{d}_{i,{\rm{intra}}})/\max({d}_{i,{\rm{intra}}},{d}_{i,{\rm{inter}}})$, where *d*_*i*,intra_ was the average distance of this unit with other units in the same cluster and *d*_*i*,inter_ was the average distance between this unit and units in the nearest cluster. The silhouette score of a clustering scheme was the average silhouette score of all units. A higher silhouette score meant a better clustering. We computed the silhouette score for the number of clusters ranging from 3 to 40. The optimal number of clusters *k* was determined by choosing the *k* with the highest silhouette score. Clustering results were robust to clustering method and to the network hyperparameters that we explored.

### Lesions

We lesioned a network unit by setting its projection weights to zero for all recurrent and output units. When we lesioned a particular network cluster, we lesioned all units within that cluster. Units were lesioned for the entire duration of the trial.

When fixed points were identified for a particular cluster lesion, we lesioned all units in the cluster and ran Fixed Point Finder^28^ for the inputs associated with the task period of interest.

### Transfer learning

Networks were pre-trained on a subset of tasks as described previously, where *W*_in_, *W*_rec_, *W*_out_ and bias vectors ${\bf{b}}_{{\rm{in}}}$, ${\bf{b}}_{{\rm{out}}}$ were learned over the course of training (see 'Training procedure' for details). After this initial stage of training, the network was trained on a held-out task. In this second phase of training, all network connections were held fixed except for the rule input weights of the held-out task. That meant that, in the second phase of learning, only a vector ${\bf{u}}^{\ast }_{{\rm{rule}}}$ of size *N*_rec_ within *W*_in_ changed.

In Extended Data Fig. 10, we wanted to understand the relationship between how well this transfer learning approach worked and whether the held-out task required learning of novel dynamical motifs. We first quantified the extent to which a task required a unique dynamical motif by comparing rows in the variance matrix (see 'Task variance analysis' for details). We sorted rows according to a reference network and computed the correlation matrix for the variance matrix in each network across all hyperparameter settings in Fig. 3b. We then took the average across all correlation matrices. We used this average correlation matrix to inform the average relationship across task periods for all networks that we examined. For each task, we identified the maximum correlation to other tasks for each task period. The most unique task period was that which had the lowest maximum correlation to other task periods. Our hypothesis was that tasks with lower correlation required unique dynamical motifs, whereas tasks with higher correlation shared dynamical motifs across tasks.

We then quantified how well our transfer learning method performed for each task. We first trained a network on all but one task in the first stage of learning and then on the held-out task in the second stage of learning. We compared the performance during training each task in the second stage of transfer learning to a single-task network. Single-task networks were trained to perform the task of interest with all weights and biases plastic. We compared the cost at two different points in the training process: (1) early in training to determine the benefit from starting with previously learned dynamical motifs and (2) late in training to determine the cost of freezing all weights except the rule input for the task of interest. We then plotted the difference in cost at these two separate timepoints against our metric for unique task periods. The fast learning benefit was smaller, and the long-term cost was negative for tasks with unique dynamical motifs.

### Statistics and reproducibility

We analyzed data from 3–5 networks with different random seeds for each experimental condition. Results were consistent across all networks that we examined; therefore, we found this sample size to be sufficient for our study. No statistical method was used to pre-determine sample size. Only networks that reached the minimum performance of 80% for every task were included in Extended Data Fig. 4. No experimental data were collected; therefore, no randomization was performed. Data analyses were not performed blinded to the conditions of network parameters.

### Reporting summary

Further information on research design is available in the Nature Portfolio Reporting Summary linked to this article.

## Online content

Any methods, additional references, Nature Portfolio reporting summaries, source data, extended data, supplementary information, acknowledgements, peer review information; details of author contributions and competing interests; and statements of data and code availability are available at 10.1038/s41593-024-01668-6.

### Supplementary information

Supplementary Table 1

Reporting Summary

## Data Availability

Trained networks were deposited on the Allen Institute database at https://codeocean.allenneuraldynamics.org/data-assets/e44a8ae2-255a-40f1-ac77-e7d04975ac8b, and additional processed data to generate all figures, including fixed point locations, were deposited at https://codeocean.allenneuraldynamics.org/data-assets/c4f26da4-73b5-4fb6-a9f2-cba52e3cf400/multitask_processed/.

## Code availability

We used publicly available GitHub repositories to identify fixed points (https://github.com/mattgolub/fixed-point-finder.git) and to generate task structures (https://github.com/gyyang/multitask.git). Custom analysis code was implemented in Python (3.10.13) and TensorFlow (2.16.1) and is publicly available (https://github.com/lauradriscoll/flexible_multitask.git).
