# Supplementary material for: Flexible multitask computation in recurrent networks utilizes shared dynamical motifs
Source: Nat Neurosci. 2024 Jul 9;27(7):1349–63. doi: 10.1038/s41593-024-01668-6 (PMC11239504; doi:10.1038/s41593-024-01668-6)
Supplement: Supplementary file 1 — Supplementary Table 1 [file 41593_2024_1668_MOESM1_ESM.pdf]

nature neuroscience

Supplementary information https://doi.org/10.1038/s41593-024-01668-6

Flexible multitask computation in recurrent networks utilizes shared dynamical motifs

In the format provided by the authors and unedited

Name:	*Description:*	*Task periods include (some or all):*	*Figures:*
**Pro stimulus**	This motif results in a response to the initial stimulus, mapping it into the same response orientation. This motif can be subdivided into Pro Delayed, Pro Integration and Categorization. All motifs within this family involve a stable fixed point in the appropriate region of state space relative to the continuous memory motif.	DelayPro[stim1], MemoryPro[stim1], ContextIntModality1[stim1], ContextIntModality2[stim1], IntegrationModality1[stim1], IntegrationModality2[stim1], IntegrationMultimodal[stim1], ReactCategoryPro[stim1], ReactMatch2Sample[stim1]	Fig.3a task period cluster 5, Ext.Data Fig.6b task period cluster 13, Ext.Data Fig.6c task period cluster 9-11
**Anti stimulus**	This motif results in a response to the initial stimulus mapping it into the opposite response orientation. This motif can be subdivided into Anti Delay and Anti Reaction. All motifs in this family involve a single stable fixed point in the appropriate region of state space relative to the continuous memory motif.	DelayAnti[stim1], MemoryAnti[stim1], ReactNonMatch2Sample[stim1], ReactCategoryAnti[stim1]	Fig.3a task period cluster 7, Ext.Data Fig.6a task period cluster 9, Ext.Data Fig.6b task period cluster 12, Ext.Data Fig.6d task period cluster 13
**Stimulus categorization**	This motif divides the initial continuous stimulus into categories based on the experimenter chosen category boundary. This motif often involves a fixed point with at least one unstable dimension nearly orthogonal to the category boundary (as shown in Fig.4e).	ReactCategoryPro[stim1], ReactCategoryAnti[stim1]	Ext.Data Fig.6a task period cluster 8, Ext.Data Fig.6b task period cluster 11 Ext.Data Fig.6d task period cluster 12
**Stimulus integration**	There are many variants of solutions for contextual integration as described in ref. 30. We often found a plane of marginally stable fixed points that integrated noisy evidence in any direction on the plane, consistent with previous work (Ext.Data Fig.8, refs 11, 30). The clusters that implement this motif can be subdivided into Modality1 Integration and Modality2 Integration.	ContextIntModality1[stim1,2], ContextIntModality2[stim1,2], IntegrationModality1[stim1,2], IntegrationModality2[stim1,2], IntegrationMultimodal[stim1,2]	Fig.3a task period cluster 4, Ext.Data Fig.6a task period cluster 4,5, Ext.Data Fig.6b task period cluster 10, Ext.Data Fig.6c task period cluster 7, Ext.Data Fig.6d task period cluster 7

**Continuous memory**	A ring of marginally stable fixed points hold the neural state in place following a stimulus period.	DelayPro[mem], MemoryPro[mem], DelayAnti[mem], MemoryAnti[mem], ContextIntModality1[mem1,2], ContextIntModality2[mem1,2], IntegrationModality1[mem1,2], IntegrationModality2[mem1,2], IntegrationMultimodal[mem1,2], ReactMatch2Sample[mem], ReactNonMatch2Sample[mem]	Fig.3a task period cluster 10, Ext.Data Fig.6a task period cluster 12, Ext.Data Fig.6b task period cluster 7-9, Ext.Data Fig.6c task period cluster 3-4, Ext.Data Fig.6d task period cluster 6
**Category memory**	Two point attractors hold the neural state in place following a stimulus period.	ReactCategoryPro[stim1], ReactCategoryAnti[stim1]	Fig.3a task period cluster 6, Ext.Data Fig.6a task period cluster 13, Ext.Data Fig.6b task period cluster 7, Ext.Data Fig.6d task period cluster 8.
**Reaction- timed response**	Stable fixed points in output potent space elicit nonzero values in the output units. This motif can be subdivided into Pro Reaction, Anti Reaction. We often additionally identified fixed points with an unstable dimension that pushes the neural state more rapidly into output potent space.	ReactPro[resp], ReactAnti[resp], ReactCategoryPro[resp], ReactCategoryAnti[resp], ReactMatch2Sample[resp], ReactNonMatch2Sample[resp]	Fig.3a task period cluster 2, Ext.Data Fig.6a task period cluster 1, Ext.Data Fig.6b task period cluster 1, Ext.Data Fig.6c task period cluster 5, Ext.Data Fig.6d task period cluster 1.
**Delayed-response**	A ring of marginally stable fixed points rotates into output potent space to elicit nonzero values in the output units. This motif is often composed of the same fixed points as the continuous memory motif, but shows up separately in the task period variance matrix because these points are in a different subspace.	DelayPro[resp], MemoryPro[resp], DelayAnti[resp], MemoryAnti[resp], ContextIntModality1[resp], ContextIntModality2[resp], IntegrationModality1[resp], IntegrationModality2[resp], IntegrationMultimodal[resp]	Fig.3a task period cluster 9, Ext.Data Fig.6a task period cluster 11, Ext.Data Fig.6b task period cluster 5, Ext.Data Fig.6c task period cluster 2, Ext.Data Fig.6d task period cluster 2.

Supplementary Table 1: Definitions of all identified motifs.
